# Identification of Fusarium head blight resistance loci in two Brazilian wheat mapping populations

**DOI:** 10.1371/journal.pone.0248184

**Published:** 2021-03-08

**Authors:** Rachel Goddard, Andrew Steed, Pedro Luiz Scheeren, João Leodato Nunes Maciel, Eduardo Caierão, Gisele Abigail Montan Torres, Luciano Consoli, Flavio Martins Santana, José Mauricio Cunha Fernandes, James Simmonds, Cristobal Uauy, James Cockram, Paul Nicholson

**Affiliations:** 1 Department of Crop Genetics, John Innes Centre, Norwich, United Kingdom; 2 Embrapa Trigo, Passo Fundo, Brazil; 3 NIAB, Cambridge, United Kingdom; Institute of Genetics and Developmental Biology Chinese Academy of Sciences, CHINA

## Abstract

Fusarium head blight (FHB) is a disease of wheat (*Triticum aestivum* L.) that causes major yield losses in South America, as well as many other wheat growing regions around the world. FHB results in low quality, contaminated grain due to the production of mycotoxins such as deoxynivalenol (DON). In Brazil, FHB outbreaks are increasing in frequency and are currently controlled by fungicides which are costly and potentially harmful to the wider environment. To identify the genetic basis of resistance to FHB in Brazilian wheat, two mapping populations (Anahuac 75 × BR 18-Terena and BR 18-Terena × BRS 179) segregating for FHB resistance were phenotyped and quantitative trait loci (QTL) analysis was undertaken to identify genomic regions associated with FHB-related traits. A total of 14 QTL associated with FHB visual symptoms were identified, each of which explained 3.7–17.3% of the phenotypic variance. Two of these QTL were stable across environments. This suggests FHB resistance in Anahuac 75, BR 18-Terena and BRS 179 is controlled by multiple genetic loci that confer relatively minor differences in resistance. A major, novel QTL associated with DON accumulation was also identified on chromosome 4B (17.8% of the phenotypic variance), as well as a major QTL associated with thousand-grain weight on chromosome 6B (16.8% phenotypic variance). These QTL could be useful breeding targets, when pyramided with major sources of resistance such as *Fhb1*, to improve grain quality and reduce the reliance on fungicides in Brazil and other countries affected by FHB.

## Introduction

Fusarium head blight (FHB) is a global disease of wheat (*Triticum aestivum* L.), causing severe epidemics in Brazil, the USA, Canada, China and Europe over recent years [1]. FHB is caused by the *Fusarium* species of hemibiotrophic fungi, with *F*. *graminearum* being the most prevalent worldwide [2]. In wheat, the characteristic symptom of FHB is the bleaching of infected spikelets prior to senescence [3]. This bleaching can spread from the initial point of infection, producing either partial or complete bleaching of the ear. FHB disease is associated with wheat yield loss, due to the development of shrivelled *Fusarium*-damaged kernels (FDK) and grain contamination due to the accumulation of mycotoxins produced by *Fusarium* fungi [4]. Due to the health risks associated with their consumption, the levels of mycotoxins, such as deoxynivalenol (DON), within grain products are strictly controlled [5].

In Brazil, the subtropical climate in the major wheat growing states of Paraná, Rio Grande do Sul and Minas Gerais provides an ideal environment for FHB development [6]. Severe FHB outbreaks are increasing in frequency, can cause yield losses of over 70% [7] and may result in increased reliance on grain imports to meet demand. A maximum DON content of 1.75 ppm in processed wheat products and 2.00 ppm in whole wheat grain is tolerated for human consumption in Brazil [8], but this is proving difficult to achieve in practice. DON was detected in 66.4% of the wheat samples collected in 2008–2009 from the Brazilian state of Paraná [9], with a mean DON content of 1.89 ppm, whilst in 2014, a year of severe FHB incidence, 58.0% of wheat samples analysed contained DON at levels higher than the maximum permissible limits [10]. Such studies demonstrate the need to reduce both the FHB incidence and mycotoxin content in Brazilian wheat grain to reduce economic losses and minimise human health risks.

Genetic resistance to FHB is polygenic and highly influenced by the environment, meaning that phenotyping is labour intensive and identifying stable sources of resistance can be difficult [11]. The most potent source of genetic resistance in wheat breeding is conferred by the *Fhb1* locus [12], identified from the Chinese cultivar Sumai 3 [13]. The nature of the underlying gene(s) is disputed and has been suggested to be a pore-forming toxin-like (*PFT*) gene [14] or a histidine-rich calcium-binding-protein gene (*His* or *HRC*) gene [15, 16]. Recent analysis has found that *Fhb1* is present at a very low frequency in Brazilian germplasm [17, 18].

Prior to the utilisation of resistance sources from Asia, such as *Fhb1*, the Brazilian spring wheat variety Frontana was widely used in Brazilian, North American and Canadian breeding programmes due to its moderate resistance [19, 20]. Resistance in Frontana appears to be conferred by multiple quantitative trait loci (QTL), some of which are coincident with QTL for morphological traits [21–23]. The association between FHB-related traits and morphological traits has also been demonstrated in European, North American and Asian wheat germplasm, with plant height and flowering time being particularly strongly associated with FHB incidence [24]. Consequently, the potential for trade-off between disease resistance and morphological characteristics must be thoroughly assessed prior to the introgression of resistance into breeding programmes.

Wheat blast, caused by the fungus *Magnaporthe oryzae Triticum* (MoT) pathotype [25], has become a severe threat to wheat production in South America and frequently occurs alongside FHB in some regions [26]. It has been suggested that resistance to FHB may result in susceptibility to wheat blast and *vice versa* [27]. For example, Sumai 3 is highly susceptible to blast whilst Milan, which carries the 2NS introgression from *Aegilops ventricosa* that confers wheat blast resistance, is highly susceptible to FHB [28]. However, it is unclear whether this resistance differential is due to pleiotropy or linkage. Wheat cultivars that display durable, broad-spectrum resistance to wheat blast, such as BR 18-Terena [29], have been widely utilised in Brazilian wheat breeding programmes to reduce disease incidence. It is therefore important to determine whether these blast resistance loci also have a pleiotropic effect on FHB susceptibility.

Here, the aims of our study were to determine the genetic basis of FHB resistance in selected Brazilian cultivars and to establish whether resistance to FHB and wheat blast are independent or potentially antagonistic. QTL analysis was performed using two bi-parental mapping populations, developed previously from Brazilian wheat cultivars showing moderate-high levels of FHB resistance (Anahuac 75 and BRS 179) crossed to the blast resistant cultivar BR 18-Terena [30]. These cultivars are present in the pedigree of several modern, elite Brazilian cultivars [31]. Both populations were phenotyped for FHB related traits (FHB visual symptoms, DON accumulation, thousand-grain weight (TGW)) and agronomic traits (plant height, flowering time), to determine the potential for trade-off between traits. We identified several genetic loci associated with FHB traits, including major QTL for resistance to DON accumulation (chromosome 4B) and TGW (chromosome 6B). We found no evidence for potential trade-off between FHB at the adult plant stage in the field and blast resistance detected previously, using detached leaf or wheat ear assays under glasshouse conditions [30]. The findings of these studies have significant implications for wheat breeding in South America and any other regions where both FHB and blast coincide.

## Materials and methods

### Plant material

The Brazilian spring wheat cultivars Anahuac 75 (I-12300//Lerma-Rojo-64/II-8156/3/Norteno-67), BRS 179 (BR 35/PF 8596/3/PF 772003*2/PF 813//PF 83899) and BR 18-Terena (pedigree unknown) [31] were used to develop two recombinant-inbred line (RIL) populations. BR 18-Terena is susceptible to FHB and was used as the common parental line in the two populations, whilst Anahuac 75 and BRS 179 display moderate-high levels of resistance. The two RIL populations, Anahuac 75 × BR 18-Terena (hereafter referred to as BR 18) and BRS 179 × BR 18, were developed to the F_6_ generation as described by Goddard et al. [30], with 188 recombinant inbred lines (RILs) per population.

### *Fusarium* phenotyping

Both populations were sown at the John Innes Centre (JIC), Norfolk, United Kingdom (52°37’20.6"N 1°13’18.3"E**)** in April 2016 and 2017. The BR 18 × BRS 179 population was also sown at Embrapa Trigo, Passo Fundo, Brazil (28°13’52.1"S 52°24’13.8"W) in August 2020. For the UK trials a split-plot design was used, with each split-plot containing a different genotype. Each trial consisted of 10 field rows containing 40 split-plots (40 genotypes) per row. For the RILs, two replicates were included per trial, and for the parental lines, eight replicates were included per trial. For the Embrapa trials, the field layout consisted of 18 field rows each containing 20 plots. Two replicates were included for both the RILs and the parental lines. Trials in both the UK and Brazil were run using standard agronomic packages of fertilisers and pesticides. Plant height, flowering time and FHB visual symptoms were recorded in each year. Height was measured as the distance from soil to the tip of the spike, excluding awns, at Zadoks growth stage GS 83 [32] and flowering time was scored as the number of days from initial sowing to GS 65 (50% of ears within each plot at mid-anthesis).

Conidial inoculum for the UK trials was produced as described by Peraldi et al. [33], using DON producing *Fusarium culmorum* isolates (Fc2037 and Fc2076) obtained from the JIC culture collection. Field plots were spray inoculated until run-off with an *F*. *culmorum* conidial suspension (0.5x10^5^ conidia per ml^-1^ and 0.05% Tween 20) from mid-anthesis (GS 65). Inoculations were repeated three times at two-day intervals, and mist irrigation was applied for 15 min following each inoculation to maintain high humidity. In the Brazil trials, the grain spawn inoculation method was used as described by Lima and Fernandes [34]. Briefly, at GS 65 *F*. *graminearum* infected wheat grains were spread around the edge of each plot. In the absence of rain, mist irrigation was applied for 5 min, nine times per day, until GS 85 (soft dough). In both the UK and Brazil trials, FHB severity (the percentage of bleached kernels in the entire plot) was scored at three separate time points beginning at 21 days post inoculation (dpi), and the area under the disease progress curve (AUDPC) was calculated.

In the UK trials, ears were hand harvested from each plot and threshed at a low wind speed to retain small, light-weight grains and remove chaff. Replicate grain samples were pooled and thousand-grain weight (TGW) was determined using a MARVIN seed analyser (GTA Sensorik GmbH, Germany). Mycotoxin analysis was undertaken on a random sub-set of harvested grain to quantify the accumulation of DON, with a minimum of 120 lines analysed per population. For each RIL, grain from the two replicates was pooled and 40g of grain was milled to provide a representative sample. For the parental lines two pooled samples were assayed per trial. DON accumulation was quantified using a Ridascreen Fast DON ELISA kit (R-Biopharm, Germany) as per the manufacturer’s instructions. DON accumulation and TGW was not assessed in the BR 18 × BRS 179 Brazil trial. Phenotype data can be found in S1 File.

### Statistical and QTL analysis

Analyses of variance (ANOVA) for phenotypic traits were conducted using a general linear model (GLM) within Genstat 20^th^ edition [35]. For the field trial data year, row and plot (within the field), replicate and genotype were included as terms within the GLM. Flowering time was also included as a covariate in the model for the FHB, TGW and DON datasets. Broad-sense heritability across trials was calculated from the variance outputs from the GLM using the equation H^2^ = σ^2^_g_/(σ^2^_g_+σ^2^_ge_/E+σ^2^_e_/rE) where σ^2^_g_ is the estimate of genetic variance, σ^2^_ge_ is the estimate of genotype × environment interaction variance, σ^2^_e_ is the estimate of residual error variance, *E* is the number of environments and *r* is the number of replicates. Predicted mean values for each RIL were generated within the GLM, for use in the QTL analysis and t-probabilities were calculated to determine significant differences between genotypes. Pearson’s correlation coefficients were calculated in Genstat to determine the correlation between FHB and the agronomic traits where measured.

Both populations were genotyped using the Axiom 35k Wheat Breeder’s Array as described by Goddard et al. [30], with the resulting genetic maps used for QTL analysis. QTL analysis was performed in Genstat using both single-trait, single-environment analysis and single-trait, multiple-environment (ME) analysis. For all analyses, a logarithm of odds (LOD) score of 3.0 was required for a QTL to be deemed significant and a mapping interval size of 5 cM was used. Initial QTL detection was performed using simple interval mapping (SIM), followed by at least two rounds of composite interval mapping (CIM) to finalise the QTL location using the candidate QTL as co-factors. A final QTL model was then fitted to produce the estimated QTL effects. For each population, QTL that were identified across trial years were classified as the same QTL if the peak markers were within 15 cM of each other. For the single-trait, multiple-environment analysis, the most appropriate variance-covariance matrix to model the correlations between the different environments was selected for each dataset, followed by SIM and CIM as described above. QTL names were assigned using standard nomenclature. For a specific trait, QTL identified on the same chromosome in both populations were given suffixes to differentiate between the QTL. ME QTL names were assigned as “ME”. From the ME analysis, only QTL that were stable across environments are presented. QTL explaining over or below 10% of the phenotypic variance were classed as major or minor QTL, respectively. The corresponding physical positions associated with the peak marker and flanking markers for each QTL were determined by aligning the marker sequences to the wheat Chinese Spring RefSeq v1.1 reference genome [36]. QTL images were produced using MapChart [37].

## Results

### Phenotyping of the Anahuac 75 × BR 18 population

**Fig 1:**
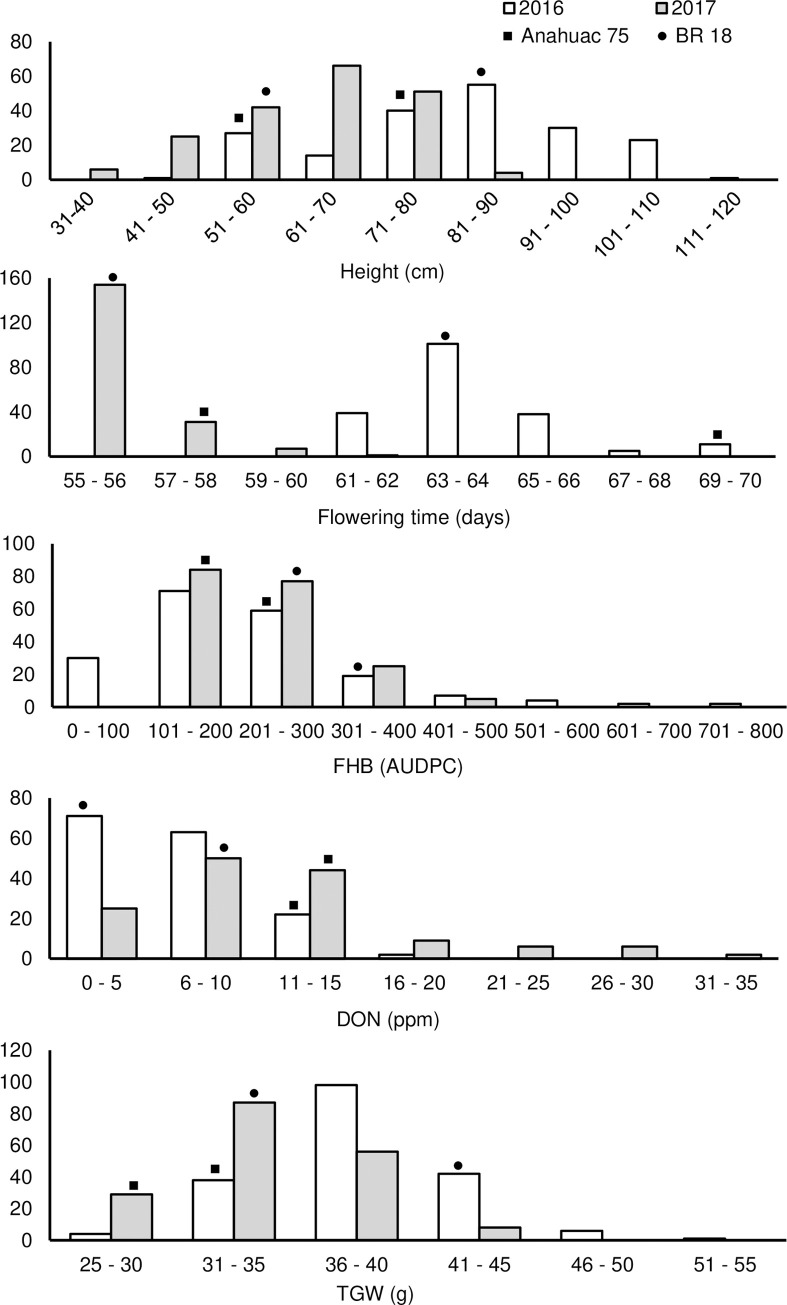
Phenotypic distributions for height, flowering time, FHB, DON accumulation and thousand-grain weight (TGW) in the Anahuac 75 × BR 18 population. The predicted mean values of the parental lines are denoted by the following symbols: ■ Anahuac 75 and ● BR 18.

BR 18 and Anahuac 75 displayed similar mean heights across trial years, however Anahuac 75 flowered consistently and significantly later than BR 18 (*P <*0.001) (Table 1). BR 18 displayed significantly more severe FHB visual symptoms than Anahuac 75 in both trial years (Table 1), however the mean DON accumulation was greater in Anahuac 75 grain samples. While this difference in DON was only significant in the 2016 trial, the same trend was observed in the 2017 trial (Table 1). The mean TGW was also lower in all Anahuac 75 samples when compared to those of BR 18, though these differences were not significant. The range of traits among the RILs are shown in Table 1. The phenotypic distributions of the predicted mean values for plant height, FHB severity (AUDPC), DON accumulation and TGW indicate transgressive segregation in the Anahuac 75 × BR 18 F_6_ population (Fig 1). For flowering time, transgressive segregation was observed only in one direction in each trial year. Broad-sense heritability estimates (*H^2^*) across environments were calculated as 0.93 for height, 0.83 for flowering time, 0.74 for FHB severity, 0.62 for DON accumulation and 0.64 for TGW.

**Table 1:** Predicted mean values from general linear modelling (GLM) of phenotypic traits for Anahuac 75 and BR 18, and the range of predicted means of the Anahuac 75 × BR 18 population.

		Parents			RILs	
Trait^a^	Year	Anahuac 75	BR 18	t- probability^b^	Mean	Range
Height	2016	77.8	81.8	0.047*	81.2	49.5–111.5
	2017	59.4	57.9	0.419	62.6	36.0–86.5
Flowering time	2016	69.0	64.0	0.001***	64.0	62.0–69.0
	2017	57.0	55.0	0.001***	56.0	55.0–61.0
FHB	2016	273.2	358.8	0.001***	215.8	37.6–770.0
	2017	191.8	258.4	0.019*	227.0	103.0–441.2
DON	2016	12.7	4.5	0.006**	6.8	0.1–18.5
	2017	10.6	9.1	0.780	11.4	0.2–34.6
TGW	2016	35.6	40.6	0.446	37.7	27.9–50.7
	2017	26.3	32.7	0.467	33.9	25.1–44.6

^a^ Height (cm), flowering time (number of days from sowing to flowering), FHB (AUDPC), DON (ppm), TGW (g).

^b^ The statistical significance of the difference between predicted mean scores for Anahuac 75 and BR 18 are shown by t-probabilities calculated within the GLM.

*, **, *** indicate *P* values of <0.05, <0.01 and <0.001, respectively.

Pearson’s correlation coefficients were calculated to determine the relationship between agronomic traits. A significant positive relationship between trial years was observed for all traits (Table 2). A significant positive correlation (≤ *P =* 0.05) was observed between height/flowering time, height/TGW and FHB/DON, in one or more trial years. Significant negative correlations (≤ *P =* 0.05) were observed between height/FHB, height/DON and FHB/TGW, in one or more years. The correlations between flowering time/FHB, flowering time/DON, flowering time/TGW and DON/TWG were both positive and negative across trial years.

**Table 2:** Pearson’s correlation coefficients calculated for phenotypic traits in the Anahuac 75 × BR 18 population.

	Height-16		Height-17		FTM-16^*ǂ*^		FTM-17^*ǂ*^		FHB-16^*ǂ*^		FHB-17^*ǂ*^		DON-16^*ǂ*^		DON-17^*ǂ*^	TGW-16^*ǂ*^		TGW-17^*ǂ*^
**Height-16**	**-**																	
**Height-17**	0.918	***	-															
**FTM-16**	0.340	***	0.274	**	-													
**FTM-17**	0.142		0.069		0.813	***	-											
**FHB-16**	-0.079		-0.105		0.525	***	0.534	***	-									
**FHB-17**	-0.534	***	-0.540	***	-0.152		0.005		0.335	***	-							
**DON-16**	-0.173		-0.172		0.283	**	0.237	*	0.504	***	0.344	***	-					
**DON-17**	-0.260	**	-0.262	**	-0.083		-0.020		0.207	*	0.404	***	0.396	***	-			
**TGW-16**	0.248	**	0.261	**	-0.204	*	-0.237	*	-0.336	***	-0.140		-0.184		0.087	-		
**TGW-17**	0.376	***	0.378	***	0.254	**	0.135	^ ^	-0.045	^ ^	-0.379	***	-0.019	^ ^	-0.102	0.356	***	-

*ǂ* FTM: flowering time, FHB: area under disease progress curve (AUDPC), DON: DON accumulation, TGW: thousand-grain weight.

*, **, *** Significantly different from zero at *P* <0.05, *P* <0.01 and *P* <0.001 level.

### QTL identified in the Anahuac 75 × BR 18 population

The 35K wheat breeder’s chip was used to genotype 188 individuals from the Anahuac 75 × BR18 population. A genetic map containing 1779 markers across 21 linkage groups was produced for QTL analysis, with 767, 739 and 273 markers located on the A, B and D genomes, respectively [30]. Markers were anchored to the wheat RefSeq v1.1 reference genome to provide physical map positions [30]. In the 2016/2017 datasets, two major height QTL were identified on the short arm of chromosomes 4B and 4D (Table 3), corresponding to the positions of the *Reduced Height* (*Rht*) semi-dwarfing genes *Rht-B1* and *Rht-D1*, respectively. Anahuac 75 contributed the *Rht-B1b* allele for reduced plant height, whilst BR 18 contributed the *Rht-D1b* short height allele. A major flowering time QTL was found on the long arm of chromosome 5B in both years and corresponds to the position of the major vernalization gene *Vrn-B1*. This QTL explained up to 36.9% of the population variance and BR 18 conferred the earlier flowering allele (Table 3). Three QTL associated with FHB were identified on chromosomes 1D, 4B and 5B, explaining 7.2–17.3% of the phenotypic variance. The chromosome 5B FHB QTL, with BR 18 conferring the resistant allele, co-located with the major chromosome 5B flowering time QTL in which Anahuac 75 conferred the late flowering allele (Table 3). Four QTL associated with DON accumulation were identified, on chromosomes 3B, 4D, 5B and 7A, explaining up to 15.1% of the variance. QTL associated with TGW were identified on chromosomes 2D, 4B and 7A.

**Table 3:** QTL identified from single-trait, single-environment QTL analysis in the Anahuac 75 × BR 18 population.

QTL^a^	Year	Peak marker	Chr^b^	Position (cM) ^c^	Interval (cM)	RefSeq (bp)^d^	LOD	% Var.^e^	Add. ^f^	s.e.^g^	Allele^h^
*QPht*.*jic-4B*.1*	2016	AX-94685096	4B	42.5	40.0–45.0	31,875,304	33.1	34.9	9.0	0.599	BR 18
*QPht*.*jic-4D*.*1*^ǂ^	2016	AX-95018920	4D	23.1	15.1–35.8	26,481,498	42.6	57.1	11.5	0.629	Anahuac 75
*QPht*.*jic-4B*.1*	2017	AX-94615340	4B	40.9	34.4–47.4	31,707,097	27.3	28.7	6.1	0.471	BR 18
*QPht*.*jic-4D*.*1*^ǂ^	2017	AX-95018920	4D	23.1	10.3–40.1	26,481,498	41.4	59.5	8.8	0.494	Anahuac 75
*QFtm*.*jic-5B*	2016	AX-95166397	5B	200.7	196.9–204.4	580,103,322	18.5	36.9	1.1	0.106	Anahuac 75
*QFtm*.*jic-2A*	2017	AX-95204097	2A	235.8	167.7–293.2	319,045,718	3.3	5.5	0.3	0.090	BR 18
*QFtm*.*jic-5B*	2017	AX-94708444	5B	195.6	186.3–204.9	572,393,698	10.8	26.5	0.6	0.094	Anahuac 75
*QFhb*.*jic-5B*	2016	AX-94814963	5B	203.0	188.7–217.3	580,686,304	5.6	11.8	43.7	9.003	Anahuac 75
*QFhb*.*jic-1D*	2017	AX-95208507	1D	260.7	229.7–291.7	458,887,427	4.0	7.2	19.7	4.919	BR 18
*QFhb*.*jic-4D*	2017	AX-94773648	4D	8.3	0.0–37.4	25,987,137	8.0	17.3	30.4	5.659	BR 18
*QFhb*.*jic-5B*	2017	AX-95108292	5B	209.1	194.3–237.9	598,078,713	4.5	9.2	22.3	5.000	Anahuac 75
*QDon*.*jic-5B*	2016	AX-94978555	5B	162.0	131.6–192.4	545,428,968	4.7	9.1	1.1	0.300	Anahuac 75
*QDon*.*jic-7A*.*1*	2016	AX-94435006	7A	77.5	65.2–89.7	670,814,974	5.8	15.1	1.4	0.311	BR 18
*QDon*.*jic-3B*	2017	AX-94684556	3B	173.3	157.2–189.5	719,085,213	4.3	10.8	2.2	0.519	Anahuac 75
*QDon*.*jic-4D*	2017	AX-94397932	4D	77.9	63.9–77.9	489,753,300	4.7	12.0	2.3	0.522	BR 18
*QTgw*.*jic-4B*	2016	AX-94508980	4B	34.8	15.6–54.0	27,522,453	4.8	9.6	1.2	0.276	BR 18
*QTgw*.*jic-7A*.*1*	2016	AX-94702814	7A	56.3	40.5–87.7	610,929,716	3.2	5.6	0.9	0.280	Anahuac 75
*QTgw*.*jic-2D*	2017	AX-94501139	2D	120.4	105.3–120.4	61,983,976	5.9	11.3	1.3	0.256	BR 18
*QTgw*.*jic-7A*.*1*	2017	AX-94763718	7A	59.3	46.4–72.2	612,990,658	6.3	12.7	1.4	0.261	Anahuac 75

^a^ QTL: *Pht*: height, *Ftm*: flowering time, *Fhb*: Fusarium head blight, *Don*: deoxynivalenol, *Tgw*: thousand-grain weight

^b^ Chr: chromosome

^c^ Position (cM): peak marker position [30]

^d^ RefSeq (bp): peak marker position in RefSeq assembly

^e^ % Var: % phenotypic variance

^f^ Add.: additive effect

^g^ s.e.: standard error

^h^ Allele: high value allele.

* QTL represents allelic variation at *Rht-B1*.

^ǂ^ QTL represents allelic variation at *Rht-D1*.

Single-trait, multiple-environment (ME) QTL analysis was undertaken on both the UK trial datasets to identify QTL that were stable across both years, and therefore not subject to a genotype × environment interaction. The major height QTL on chromosomes 4B and 4D were identified from the ME analysis, as were the flowering time and FHB QTL on chromosome 5B (Table 4). None of the QTL associated with DON were identified from the ME analysis, suggesting all four DON QTL display a genotype × environment interaction. The TGW QTL on chromosome 7A was identified from the ME analysis, suggesting it is stable across environments (Table 4). All QTL images are presented in S1–S8 Figs.

**Table 4:** Stable QTL identified from single-trait, multiple-environment QTL analysis in the Anahuac 75 × BR 18 population.

QTL^a^	Peak marker	Chr^b^	Position (cM)^c^	Interval (cM)	RefSeq (bp)^d^	LOD	% Var.^e^	Add.^f^	s.e.^g^	Allele^h^
*QME*.*Pht*.jic-4B*	AX-94685096	4B	42.5	39.5–45.2	31,875,304	66.2	36.6	9.2	0.531	BR 18
*QME*.*Pht*.*jic-4D*.*1*^ǂ^	AX-95018920	4D	23.1	19.1–35.9	26,481,498	79.8	62.8	11.7	0.556	Anahuac 75
*QME*.*Ftm*.*jic-5B*	AX-95166397	5B	200.7	196.7–204.7	580,103,322	25.6	34.5	1.0	0.095	Anahuac 75
*QME*.*Fhb*.*jic-5B*	AX-94814963	5B	203.0	187.8–218.2	580,686,304	8.3	11.3	42.7	7.269	Anahuac 75
*QME*.*Tgw*.*jic-7A*.*1*	AX-94763718	7A	59.3	46.0–75.0	612,990,658	5.5	7.9	1.1	0.230	Anahuac 75

^a^ QTL: *Pht*: height, *Ftm*: flowering time, *Fhb*: Fusarium head blight, *Don*: deoxynivalenol, *Tgw*: thousand-grain weight

^b^ Chr: chromosome

^c^ Position (cM): peak marker position [30]

^d^ RefSeq (bp): peak marker position in RefSeq assembly

^e^ % Var: % phenotypic variance

^f^ Add.: additive effect

^g^ s.e.: standard error

^h^ Allele: high value allele.

* QTL represents allelic variation at *Rht-B1*.

^ǂ^ QTL represents allelic variation at *Rht-D1*.

### Phenotyping of the BR 18 × BRS 179 population

**Fig 2:**
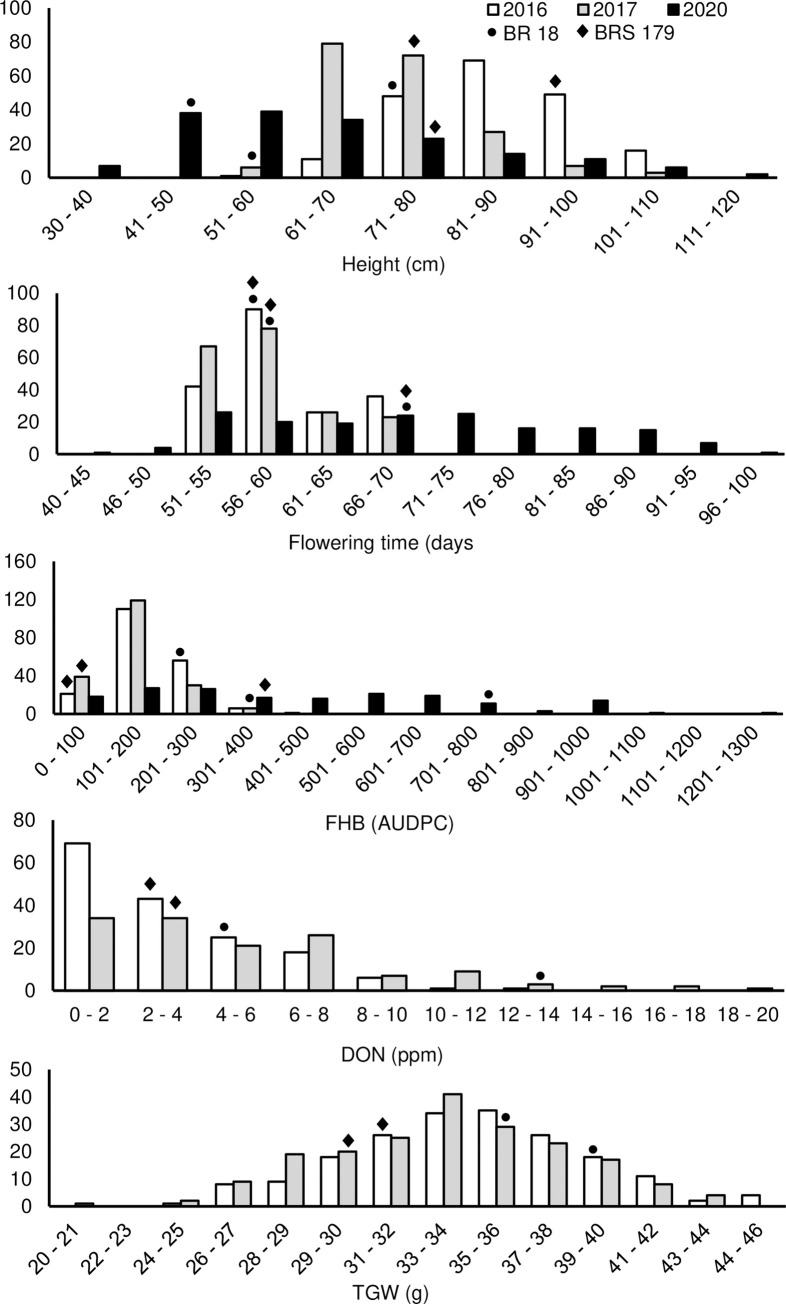
Phenotypic distributions for height, flowering time, FHB, DON accumulation and thousand-grain weight (TGW) in the BR 18 × BRS 179 population. The predicted mean values of the parental lines are denoted by the following symbols: ● BR 18 and ♦ BRS 179.

In all three trial years BRS 179 was significantly taller than BR 18 (Table 5). However, there was no significant difference in flowering time between the parental lines in the three environments. BRS 179 was significantly more resistant to FHB than BR 18 across all environments (*P* <0.001), however disease levels were much higher in the Brazil trial for both parents. The mean DON content of BRS 179 was significantly lower than in BR 18 in only a single year (Table 5). The mean TGW was also lower in all BR 18 samples when compared to those of BRS 179, however these differences were not significant. The phenotypic distributions of the predicted mean values in the BR 18 × BRS 179 F_6_ RIL population for all traits indicate transgressive segregation in both directions (Fig 2). In comparison to the UK trials, the phenotypic distribution of plant height, flowering time and FHB severity in the RIL population was greatly increased in the Brazil trial. Broad-sense heritability estimates (*H^2^*) across environments were calculated as 0.80 for height, 0.85 for flowering time, 0.67 for FHB severity, 0.66 for DON accumulation and 0.66 for TGW.

**Table 5:** Predicted mean values from general linear modelling (GLM) of phenotypic traits for BR 18 and BRS 179, and the range of predicted means of the BR 18 × BRS 179 F_6_ RILs.

		Parents				RILs	
Trait^a^	Year	BR 18	BRS 179	t- probability^b^		Mean	Range
Height	2016	79.1	97.7	0.001	***	85.8	59.5–107.0
	2017	59.8	77.2	0.001	***	72.8	54.5–103.5
	2020	45.0	80.0	0.001	***	64.6	34.9–120.0
Flowering time	2016	60.0	60.0	0.477		60.0	53.0–69.0
	2017	60.0	60.0	0.477		58.0	55.0–69.0
	2020	67.0	70.0	0.482		69.0	41.0–97.0
FHB	2016	213.9	71.3	0.001	***	173.4	48.1–409.5
	2017	320.6	81.0	0.001	***	152.8	28.4–392.7
	2020	746.4	407.3	0.001	***	433.8	41.0–1260.0
DON	2016	5.5	3.0	0.243		3.5	0.0–12.8
	2017	13.0	2.8	0.015	*	5.4	0.1–19.4
TGW	2016	39.3	30.5	0.051		34.7	25.4–45.4
	2017	35.9	29.1	0.235		34.2	20.3–43.4

^a^ Height (cm), flowering time (number of days from sowing to flowering), FHB (AUDPC), DON (ppm), TGW (g).

^b^ The statistical significance of the difference between predicted mean scores for Anahuac 75 and BR 18 are shown by t-probabilities calculated within the GLM.

*, *** indicate *P* values of <0.05 and <0.001, respectively.

As with the Anahuac 75 × BR 18 population, a significant positive relationship between trial years was observed for all traits, where measured, in the BR 18 × BRS 179 population (Table 6). Significant positive correlations (≤ *P =* 0.05) were observed between height/flowering time, FHB/DON and flowering time/DON, in one or more trial years. A very weak positive correlation was observed for DON/TGW. Significant negative correlations (≤ *P =* 0.05) were observed between height/FHB and flowering time/FHB, in one or more year. Correlations between height/DON, flowering time/TGW and FHB/TGW were negative, but not significant. The correlation between height/TGW were both positive and negative across trial years.

**Table 6:** Pearson’s correlation coefficients calculated for phenotypic traits in the BR 18 × BRS 179 population.

	Height-16		Height-17		Height-20		FTM-16^*ǂ*^		FTM-17^*ǂ*^		FTM-20^*ǂ*^		FHB-16^*ǂ*^		FHB-17^*ǂ*^		FHB-20^*ǂ*^	DON-16^*ǂ*^		DON-17^*ǂ*^	TGW-16^*ǂ*^		TGW-17^*ǂ*^
**Height-16**	-																						
**Height-17**	0.728	***	-																				
**Height-20**	0.550	***	0.575	***	-																		
**FTM-16**	0.206	*	0.446	***	0.284	**	-																
**FTM-17**	0.121		0.424	***	0.245	**	0.901	***	-														
**FTM-20**	0.153		0.264	**	0.625	***	0.472	***	0.443	***	-												
**FHB-16**	-0.117		-0.286	**	-0.191	*	-0.312	***	-0.327	***	-0.325	***	-										
**FHB-17**	-0.151		-0.054		-0.135		-0.115		-0.168		-0.143		0.255	**	-								
**FHB-20**	-0.238	**	-0.254	**	-0.585	***	-0.403	***	-0.363	***	-0.700	***	0.296	**	0.139		-						
**DON-16**	-0.130		0.003		-0.033		0.237	**	0.230	*	0.068		0.102		0.237	*	-0.040	-					
**DON-17**	-0.213	*	0.030		-0.037		0.429	***	0.384	***	0.233	*	0.002		0.374	***	-0.117	0.513	***	-			
**TGW-16**	0.215		0.215	*	0.301	***	-0.016		-0.075		0.099		0.043		0.047		-0.063	0.023		0.094	-		
**TGW-17**	-0.025		-0.022		-0.049		-0.117	^ ^	-0.119	^ ^	-0.106	^ ^	-0.038		-0.068		0.021	0.150		0.030	0.568	***	-

*ǂ* FTM: flowering time, FHB: area under the disease progress curve (AUDPC), DON: DON accumulation, TGW: thousand-grain weight.

*, **, *** Significantly different from zero at *P* <0.05, *P* <0.01 and *P* <0.001 level.

### QTL identified in the BR 18 × BRS 179 population

The 35K wheat breeder’s chip was used to genotype 188 individuals from the BR 18 × BRS 179 population. A genetic map containing 1318 markers across 21 linkage groups was produced for QTL analysis, with 690, 556 and 72 markers located on the A, B and D genomes, respectively [30]. Markers were anchored to the wheat RefSeq v1.1 reference genome to provide physical map positions [30]. In this population, height QTL were identified on chromosomes 1A, 2D, 4B, 4D, 6A, 6B and 7A (Table 7). The major chromosome 4D QTL, corresponding to the position of the *Rht*-*D1* semi-dwarfing gene, explained up to 39.1% of the phenotypic variance, with BR 18 contributing the *Rht-D1b* semi-dwarfing allele. Flowering time QTL were identified on chromosomes 1A, 2A, 2B, 2D, 6B and 7A (Table 7). The major QTL (*QFtm*.*jic-7A*.*1*) conferred up to 41.2% of the variance and corresponds to the position of the flowering time gene *Vrn-A3*. BR 18 provided the late flowering allele at this locus. FHB associated QTL were identified on chromosomes 1A, 2A, 2D, 3B, 4A and 7A, and conferred up to 14.8% of the variance. BRS 179 provided the resistant allele at all loci except chromosomes 3B and 4A (Table 7). Four QTL associated with DON accumulation were identified, on chromosomes 2A, 4B, 6A and 7A, with BRS 179 conferring the low DON allele at all loci (Table 7). The DON QTL on chromosome 4B was identified in both trial years, explaining up to 14.9% of the variance. TGW QTL were identified on chromosomes 6B and 7A in both trial years (Table 7).

**Table 7:** QTL identified from single-trait, single-environment QTL analysis in the BR 18 × BRS 179 population.

QTL^a^	Year	Peak marker	Chr^b^	Position (cM)	QTL interval (cM)	RefSeq position (bp)	LOD	% Var.^c^	Add.^d^	s.e.	Allele^e^
*QPht*.*jic-4B*.*2*	2016	AX-94448564	4B	132.8	0.0–169.1	653,525,789	3.3	4.0	2.1	0.582	BRS 179
*QPht*.*jic-4D*.*2*	2016	AX-94728173	4D	9.1	5.6–12.7	3,142,500	21.6	39.1	6.5	0.582	BRS 179
*QPht*.*jic-6A*	2016	AX-94586966	6A	59.7	37.0–82.3	37,415,350	6.3	8.7	3.1	0.589	BR 18
*QPht*.*jic-4D*.*2*	2017	AX-94728173	4D	9.1	0.0–16.5	3,142,500	8.3	16.7	3.8	0.625	BRS 179
*QPht*.*jic-6A*	2017	AX-94590712	6A	68.2	35.8–95.1	63,785,177	4.1	7.1	2.5	0.622	BR 18
*QPht*.*jic-1A*	2020	AX-94395420	1A	192.2	168.1–199.0	570,450,127	4.0	11.2	6.3	1.572	BRS 179
*QPht*.*jic-2D*	2020	AX-94615229	2D	41.1	37.6–53.6	20,925,377	6.9	16.1	7.5	1.367	BRS 179
*QPht*.*jic-4D*.*2*	2020	AX-94547815	4D	0.0	0.0–16.5	14,986,403	4.8	8.1	5.4	1.195	BRS 179
*QPht*.*jic-6B*	2020	AX-94850241	6B	93.7	81.7–119.3	663,800,610	3.6	5.7	4.5	1.206	BR 18
*QPht*.*jic-7A*	2020	AX-94398969	7A	83.8	34.6–132.9	76,124,500	3.8	6.0	4.6	1.197	BR 18
*QFtm*.*jic-7A*.*1*	2016	AX-94398969	7A	83.8	80.4–87.1	76,124,550	22.2	41.2	2.9	0.253	BR 18
*QFtm*.*jic-7A*.*2*	2016	AX-95248570	7A	177.8	0.0–197.6	701,360,939	3.3	4.0	0.9	0.253	BRS 179
*QFtm*.*jic-7A*.*1*	2017	AX-94906538	7A	84.3	77.6–91.1	76,504,019	10.9	21.8	1.9	0.258	BR 18
*QFtm*.*jic-7A*.*2*	2017	AX-95248570	7A	177.8	0.0–197.6	701,360,939	3.3	5.2	0.9	0.258	BRS 179
*QFtm*.*jic-1A*	2020	AX-94674333	1A	163.6	138.8–188.4	551,607,501	5.5	8.2	3.7	0.763	BRS 179
*QFtm*.*jic-2A*	2020	AX-94900000	2A	47.3	14.9–73.3	27,981,585	4.6	7.5	3.5	0.812	BR 18
*QFtm*.*jic-2B*	2020	AX-94459800	2B	16.5	0.0–47.9	52,911,848	4.7	7.2	3.5	0.784	BR 18
*QFtm*.*jic-2D*	2020	AX-94563255	2D	53.6	28.4–53.6	27,924,890	5.1	8.1	3.7	0.796	BRS 179
*QFtm*.*jic-6B*	2020	AX-94480407	6B	80.1	60.3–100.1	566,541,021	3.1	4.3	2.7	0.782	BR 18
*QFtm*.*jic-7A*.*1*	2020	AX-94906538	7A	84.3	66.5–103.7	76,504,019	6.4	9.8	4.0	0.760	BR 18
*QFhb*.*jic-2A*.*1*	2016	AX-95227146	2A	4.9	0.0–43.7	3,334,456	4.5	6.5	16.3	4.448	BR 18
*QFhb*.*jic-3B*	2016	AX-95230609	3B	110.0	0.0–120.7	676,617,701	3.0	3.7	0.6	0.186	BRS 179
*QFhb*.*jic-7A*.*1*	2016	AX-94472422	7A	2.0	0.0–67.2	8,383,300	3.5	5.8	10.3	4.467	BR 18
*QFhb*.*jic-2A*.*2*	2017	AX-94513872	2A	28.9	0.0–57.3	15,589,919	4.7	8.6	18.3	4.578	BR 18
*QFhb*.*jic-4A*	2017	AX-94720747	4A	24.4	6.6–42.2	544,390,969	5.4	10.1	19.9	4.171	BRS 179
*QFhb*.*jic-7A*.*1*	2017	AX-94458095	7A	9.5	0.0–51.3	9,158,773	3.5	5.8	14.7	4.186	BR 18
*QFhb*.*jic-1A*	2020	AX-94395420	1A	192.2	162.4–207.4	570,450,127	3.2	8.9	87.1	25.132	BR 18
*QFhb*.*jic-2D*	2020	AX-94563255	2D	53.6	44.6–53.6	27,924,890	7.2	14.8	106.7	18.958	BR 18
*QFhb*.*jic-7A*.*1*	2020	AX-94826773	7A	17.0	0.0–48.0	20,768,360	3.2	7.6	70.7	18.869	BR 18
*QFhb*.*jic-7A*.*2*	2020	AX-94454991	7A	84.3	57.5–112.8	76,504,019	3.5	8.9	77.0	18.478	BR 18
*QDon*.*jic-2A*	2016	AX-94930415	2A	152.2	140.3–164.1	509,357,751	5.8	13.6	1.0	0.201	BR 18
*QDon*.*jic-4B*	2016	AX-94657860	4B	90.7	82.3–108.2	574,059,204	6.5	14.8	1.0	0.196	BR 18
*QDon*.*jic-4B*	2017	AX-95652956	4B	80.3	69.6–91.0	535,049,553	6.6	14.9	1.5	0.276	BR 18
*QDon*.*jic-6A*	2017	AX-94882086	6A	94.5	74.2–95.1	581,894,045	4.4	9.3	1.2	0.279	BR 18
*QDon*.*jic-7A*.*2*	2017	AX-94398361	7A	111.3	78.1–144.6	617,684,953	3.5	7.0	1.0	0.277	BR 18
*QTgw*.*jic-6B*	2016	AX-94399591	6B	62.7	54.2–71.2	140,849,796	9.3	17.9	1.8	0.266	BR 18
*QTgw*.*jic-7A*.*2*	2016	AX-95081294	7A	122.4	82.7–131.2	652,689,743	4.1	7.1	1.1	0.272	BRS 179
*QTgw*.*jic-6B*	2017	AX-94695383	6B	58.9	47.0–70.7	118,186,233	5.7	10.3	1.6	0.278	BR 18
*QTgw*.*jic-7A*.*2*	2017	AX-94398361	7A	111.3	94.0–128.6	617,684,953	8.0	13.7	1.4	0.262	BRS 179

^a^ QTL: *Pht*: height, *Ftm*: flowering time, *Fhb*: Fusarium head blight, *Don*: deoxynivalenol, *Tgw*: thousand-grain weight

^b^ Chr: chromosome

^c^ Position (cM): peak marker position [30]

^d^ RefSeq (bp): peak marker position in RefSeq assembly

^e^ % Var: % phenotypic variance

^f^ Add.: additive effect

^g^ s.e.: standard error

^h^ Allele: high value allele.

^ǂ^ QTL represents allelic variation at *Rht-D1*.

**Fig 3:**
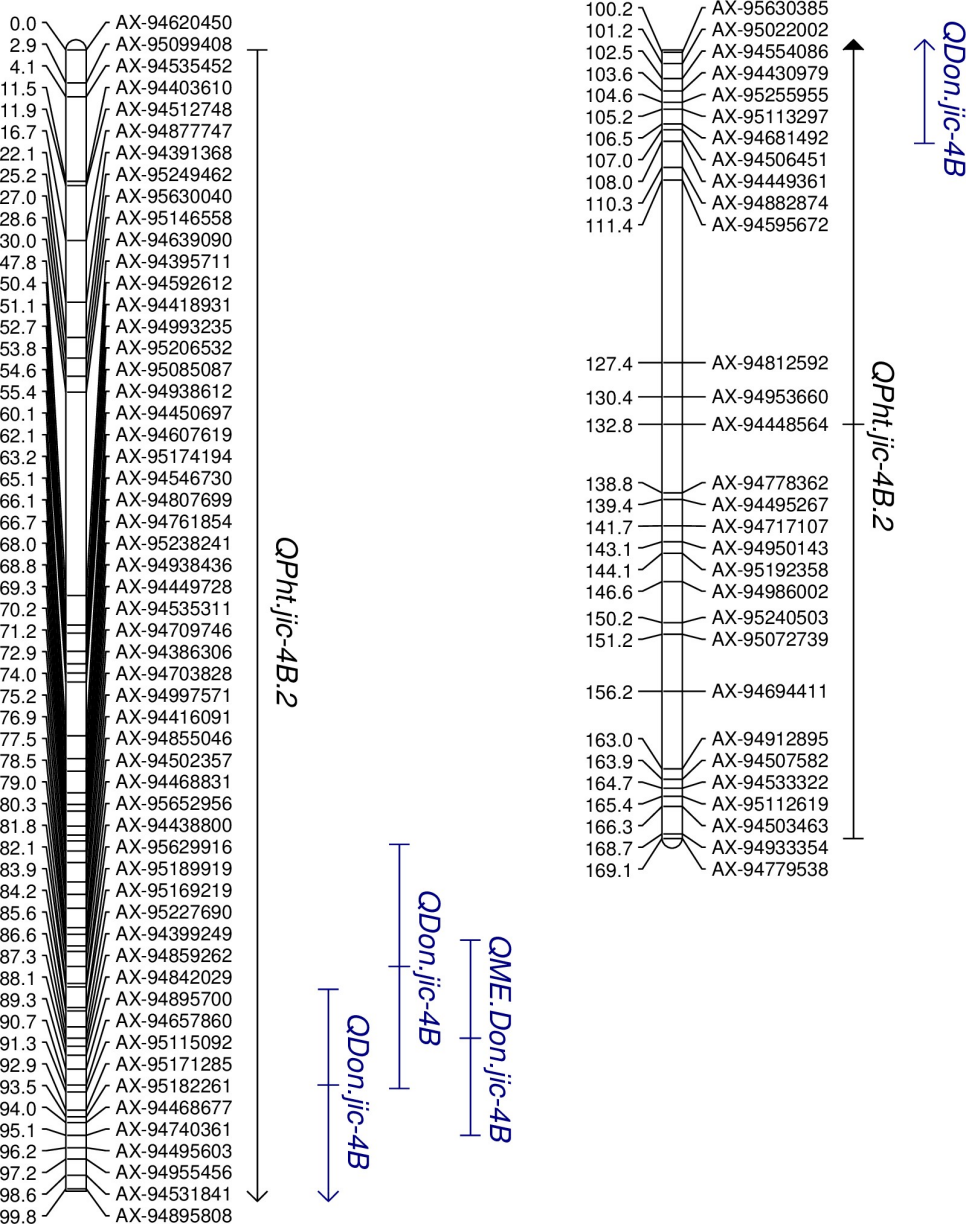
QTL associated with DON accumulation and plant height on chromosome 4B in the BR 18 × BRS 179 F_6_ RIL population.

Both the major chromosome 4D and minor chromosome 6A height QTL were determined to be stable across all three environments, (Table 8), as were both of the 7A flowering time QTL. The FHB QTL on chromosome 7A was also stable across the three environments. The chromosome 4B DON accumulation QTL was identified at the consensus position of 86.6 cM (Table 8, Fig 3). The TGW QTL on chromosomes 6B and 7A were also identified in the ME analysis (Table 8). All QTL images are presented in S9–S18 Figs.

**Table 8:** Stable QTL identified from single-trait, multiple-environment QTL analysis in the BR 18 × BRS 179 population.

QTL^a^	Peak marker	Chr^b^	Position (cM)	QTL interval (cM)	RefSeq position (bp)	LOD	% Var.^c^	Add.^d^	s.e.	Allele^e^
*QME*.*Pht*.*jic-4D*.*2*^ǂ^	AX-94728173	4D	9.1	5.4–12.9	3,142,500	23.0	37.2	6.3	0.608	BRS 179
*QME*.*Pht*.*jic-6A*	AX-94586966	6A	59.7	28.8–90.5	37,415,350	5.1	6.0	2.5	0.567	BR 18
*QME*.*Ftm*.*jic-7A*.*1*	AX-94398969	7A	83.8	80.2–87.4	76,124,500	32.2	38.3	2.8	0.254	BR 18
*QME*.*Ftm*.*jic-7A*.*2*	AX-95248570	7A	177.8	0.0–197.6	701,360,939	3.8	5.4	0.9	0.245	BRS 179
*QME*.*Fhb*.*jic-7A*	AX-94496922	7A	8.1	0.0–40.1	7,586,807	4.5	5.6	13.4	3.239	BRS 179
*QME*.*Don*.jic-4B*	AX-94399249	4B	86.6	78.0–95.1	539,505,292	11.4	17.8	1.6	0.231	BR 18
*QME*.*Tgw*.jic-6A*	AX-94695383	6B	58.9	49.7–70.1	118,186,233	13.4	16.8	1.7	0.224	BR 18
*QME*.*Tgw*.*jic-7A*.2*	AX-94398361	7A	111.3	101.3–121.4	617,684,953	9.1	15.6	1.7	0.264	BRS 179

^a^ QTL: *Pht*: height, *Ftm*: flowering time, *Fhb*: Fusarium head blight, *Don*: deoxynivalenol, *Tgw*: thousand-grain weight

^b^ Chr: chromosome

^c^ Position (cM): peak marker position [30]

^d^ RefSeq (bp): peak marker position in RefSeq assembly

^e^ % Var: % phenotypic variance

^f^ Add.: additive effect

^g^ s.e.: standard error

^h^ Allele: high value allele.

^ǂ^ QTL represents allelic variation at *Rht-D1*.

* QTL identified from UK trial data.

## Discussion

Phenotyping and QTL analysis of FHB is difficult due to the complex interaction between environmental factors and host developmental characters making FHB QTL less easy to define than those for highly heritable traits. In this study the broad-sense heritability estimates (*H^2^*) for agronomic traits, such as plant height and flowering time, were high whilst those for FHB associated traits (visual severity, DON accumulation) were more moderate. These broad-sense heritability estimates for both types of traits are comparable with *H^2^* values previously reported in the literature [38, 39]. In the present study, a single FHB QTL per population was stable across environments. BRS 179 carried an FHB resistance allele at the QTL on chromosome 5B with moderate effect (*QME*.*Fhb*.*jic-5B*, 11.3% variance) while BR 18 carried a resistance allele of lesser effect at the chromosome 7A QTL (*QME*.*Fhb*.*jic-7A*, 5.6% variance). These findings suggest that FHB resistance in the Brazilian cultivars Anahuac 75, BR 18 and BRS 179 is controlled by multiple genetic loci that confer quantitative differences in resistance.

The peak marker for *QME*.*Fhb*.*jic-5B* was found within 1.00 Mb of the major flowering time QTL (34.5% variance) on the long arm of chromosome 5B (*QME*.*Ftm*.*jic-5B*), with peak markers in the wheat reference genome Chinese Spring located at 580.10 Mb and 580.68 Mb, respectively [36]. The overall correlation between flowering time and FHB in this population, however, was environmentally dependent, with both positive and negative correlation coefficients between the two traits across trial years. The effect of the chromosome 5B QTL on the two traits, however, was stable across years with the BR18 allele being associated with both early flowering and reduced FHB symptoms.

The flowering time QTL identified in the population may reflect allelic differences between Anahuac 75 and BR 18 within the wheat vernalisation gene *Vrn-B1*. *Vrn-B1* is present on the long arm of chromosome 5B (gene model *TraesCS5B02G396600*, located at 573.80 Mb) within the *QME*.*Ftm*.*jic-5B* interval (572.39–584.88 Mb) and is homologous to the Arabidopsis *APETALA 1* (*AP1*) gene [40]. The presence of dominant early flowering *Vrn-B1* alleles determines a ‘spring’ growth habit [40]. Several FHB QTL have been identified near *Vrn-B1* [41–43], suggesting this region has a strong association with FHB resistance. FHB QTL identified in this region have also shown associations with traits such as plant height, flowering time, DON and FDK, in both European winter wheat and Brazilian spring wheat backgrounds [23, 43]. This suggests that *Vrn-B1* may have a pleiotropic effect on FHB severity and other related traits. Alternatively, there may be multiple, closely linked genes associated with different traits within this chromosome 5B long arm region.

Whilst *QME*.*Fhb*.*jic-5B*, identified in the Anahuac 75 × BR 18 population, appears to have an association with flowering time, the stable QTL *QME*.*Fhb*.*jic-7A* does not display an association with any of the agronomic traits investigated here. *QME*.*Fhb*.*jic-7A* is located on the short arm of chromosome 7A (peak marker at 7.58 Mb) in the BR 18 × BRS 179 population, with BRS 179 providing the resistant allele. This QTL appears to be solely associated with FHB visual symptoms. FHB-associated QTL have been identified on the short of 7A in hexaploid, synthetic and durum (*Triticum turgidum* L. var. *durum*) wheat [44]. Within the centromeric region on the short arm of chromosome 7A, Ruan et al. [45] identified the QTL *QFhb*.*usw-7A2* (9.0% variance), which was associated with FHB incidence and severity, whilst He et al. [43] identified a QTL associated with FDK but not visual symptoms (7.5% variance). Following point inoculation, which determines resistance to the spread of FHB within the wheat head (Type 2 resistance), both Zhang et al. [46] and Zhao et al. [47] identified QTL which were more distally located from the centromere. The physical position of the peak marker of the QTL *QME*.*Fhb*.*jic-7A* we identified here is located much closer to the telomeric end of the short arm of chromosome 7A than any of the previously reported QTL, suggesting it is likely to be novel. However, as the phenotypic variance conferred by *QME*.*Fhb*.*jic-7A* is under 6.0%, this is not a major breeding target on its own and would best be used alongside major resistance sources and/or other resistance QTL of minor effect.

The co-localisation of QTL for plant height and FHB severity has been observed on several wheat chromosomes [48–50], with increased height positively correlating with disease resistance. The major effect height QTL in both populations were associated with the *Rht* semi-dwarfing genes *Rht-B1* and *Rht-D1* on the short arms of chromosomes 4B and 4D, respectively. The *Rht-B1b* and *Rht-D1b* alleles at these loci confer insensitivity to the phytohormone gibberellic acid (GA) [51] and have been associated with large increases in susceptibility to FHB [50, 52]. The Anahuac 75 × BR 18 population segregates for semi-dwarfing alleles at both the *Rht-B1* (Anahuac 75) and *Rht-D1* (BR 18) loci, whilst the BR 18 × BRS 179 population only segregates for semi-dwarfing alleles at the *Rht-D1* locus, as BRS 179 possesses the wild-type ‘tall’ allele at both *Rht* loci. Surprisingly, none of the major height QTL identified in either population were associated with stable FHB QTL. Whilst both *Rht* semi-dwarfing alleles reduce plant height by 15.0–20.0% [53], *Rht-B1b* has a lesser effect on Type 1 FHB susceptibility than *Rht-D1b* [38, 54], which may explain the absence of an association between *Rht-B1b* and FHB QTL in the Anahuac 75 × BR 18 population. However, as the presence of *Rht-D1b* has been demonstrated to increase FHB susceptibility by up to 52.0% [50, 55] it is surprising that there were no strong, consistent associations observed between FHB QTL and *Rht-D1b* on chromosome 4D in either population. Several studies have suggested that the increased disease susceptibility associated with *Rht-D1b* is not due to height *per se*, but caused by potential linkage with deleterious genes [38, 50]. Whilst significant marker-trait associations between FHB and *Rht-D1b* have consistently been found in European winter wheats [56], it is possible that within Brazilian spring wheat breeding programmes the linkage with deleterious genes has been broken or is fixed irrespective of whether cultivars carry either *Rht-D1a* or *Rht-D1b* alleles.

Cereal crops are most susceptible to FHB during anthesis, particularly if the flowering period coincides with the warm, humid conditions which promote disease development. Consequently, FHB traits and flowering time QTL are often associated in mapping studies [48, 57, 58], particularly in naturally inoculated experiments. In our study, each genotype was inoculated from mid-anthesis to minimise the effect of flowering time on disease resistance. In the BR 18 × BRS 179 population, a 16-day flowering time window between the earliest and latest flowering RILs was observed in the UK trials. In the Brazil trial, this flowering time window was extended to over 50 days, which may be due to differences in photoperiod and/or temperature between the two environments. As such, one might expect a possible association between flowering time and FHB resistance, particularly given the potential for varying environmental conditions during this window. Whilst stable QTL for flowering time (38.3% variance) and FHB visual symptoms (5.6% variance) were both identified on the short arm of 7A, in both the UK and Brazil trials, these QTL did not co-locate. The peak marker for the FHB QTL, *QME*.*Fhb*.*jic-7A* (7.58 Mb), was located much closer to the telomeric region of the short arm of chromosome 7A than the peak marker of the flowering time QTL, *QME*.*Ftm*.*jic-7A*.*1* (76.12 Mb). The second flowering time QTL (5.4% variance) identified was on the long arm of chromosome 7A (peak marker at 701.36 Mb), again demonstrating a lack of association with *QME*.*Fhb*.*jic-7A*. Given the positioning of the major flowering time QTL *QME*.*Ftm*.*jic-7A*.*1* on the short arm of chromosome 7A (64.73–79.65 Mb), it is possible that this reflects the presence of different *Vrn-A3* alleles in the BR 18 × BRS 179 lines. *Vrn-A3* is found on 7AS (gene model *TraesCS7A02G115400*, located at 71.66–71.67 Mb) and has a crucial role in integrating vernalization and photoperiod signals [59, 60]. Associations between *Vrn-D3*, the D-genome homoeologue on chromosome 7D, and FHB traits (DON accumulation and FDK) have been observed previously [61], however no association between flowering time and FHB traits within the *Vrn-A3* region was seen in this study.

In Brazil, increasingly frequent FHB epidemics have led to concerns about the control of DON contamination in wheat grain [10]. DON acts as a virulence factor in wheat, enabling the *Fusarium* fungus to spread within the infected wheat head [62, 63]. Whilst DON has a key role in FHB infection, QTL associated with FHB resistance and reduced DON accumulation can be non-coincident [43, 64–66]. QTL associated with DON accumulation are often environmentally dependent [22], as demonstrated in this study where seven DON-associated QTL were identified in a single trial year only. A single major QTL, *QME*.*Don*.*jic-4B*, from the single-trait, multiple-environment QTL analysis was identified in the BR 18 × BRS 179 population. This derived from the peak markers of two DON QTL mapping within a 10.0 cM region on the long arm of chromosome 4B across two trial years. This locus appears to be particularly potent, explaining up to 17.8% of the phenotypic variance, suggesting it is a worthwhile breeding target for further investigation. Whilst several studies have identified QTL for DON on the short arm of chromosome 4B [23, 67], few QTL have been identified on the long arm. Wang et al. [68] identified significant marker-trait associations for both FHB severity and DON accumulation on the long arm of 4B in a genome wide association mapping study of CIMMYT spring wheats. The markers associated with these traits map to a physical region between 281.70–427.50 Mb on 4B, whilst the QTL region for *QME*.*Don*.*jic-4B* is found between 527.90–583.33 Mb (peak marker at 539.50 Mb). Resistance to both DON and FDK has been identified on the long arm of 4B in the winter wheat variety Ernie [69], however the QTL peak marker was located at 482.82 Mb, which is 56.68 Mb more proximal than the peak marker in our study. This suggests that the QTL observed in this study may be novel. QTL solely associated with reduced DON content are rarely identified [43]. *QME*.*Don*.*jic-4B* is not associated with QTL for FHB visual symptoms, FDK or agronomic QTL, suggesting that reduced DON accumulation within this region is not due to the pleiotropic effects of other traits or disease escape. It is possible that the 4BL QTL region may represent a gene associated with DON detoxification, such as a *UDP glucosyltransferase* (*UGT*) which glycosylates DON to DON-3-*O*-glucoside (D3G), a compound with reduced toxicity [70]. The activity of *UGT* genes have been proposed as a major mechanism of DON resistance and the expression of monocot *UGT* candidate genes has been shown to affect DON accumulation *in planta*. Constitutive expression of the barley (*Hordeum vulgare*) *UGT* gene *HvUGT13248* results in increased DON-3-*O*-glucoside production and greater Type 2 resistance in the wheat cultivar Bobwhite, both in controlled and field conditions [71]. The wheat orthologue of the *Brachypodium distachyon Bradi5g03300 UGT* gene, identified as *TraesCS2B02G068700* also provides increased tolerance to DON in the root when expressed in *B*. *distachyon* [72]. Several wheat *UGT* genes have been identified through synteny with other monocots [72, 73] and a further 179 putative *UGT* genes have been predicted by performing genome wide analysis to identify conserved domains associated with family-1 *UGT* genes [74]. Of these 179 potential genes, a single *UGT* gene on the long arm of chromosome 4B was identified near the telomeric region (663.65 Mb). The physical position of this gene indicates that the putative *UGT* identified by He et al. [74] is not associated with the DON accumulation QTL identified in our study. Several *UGT* genes, such as *TaUGT3* on 3B and *TaUGT12887* on 5A [73, 75], have been shown to have a minimal effect on increasing DON tolerance, suggesting it is possible that DON resistance on the long arm of chromosome 4B may be associated with other mechanisms. DON detoxification/resistance has also been linked with genes involved in phytohormone signalling pathways, transporter proteins and cytochrome P450 and methionyl-tRNA synthetase enzymes [76]. Further refinement of the 4B QTL region will be required to determine which genes may be potential candidates.

FHB infection is often associated with the development of shrivelled ‘tombstone’ damaged kernels which have low grain weight and may be contaminated with mycotoxins [4]. In this study, QTL associated with TGW were identified on chromosome 7A in both populations. The QTL *QME*.*Tgw*.*jic-7A*.*1* (peak marker at 612.99 Mb) was identified in the Anahuac 75 × BR 18 population, whilst *QME*.*Tgw*.*jic-7A*.*2* (peak marker at 617.68 Mb) was identified in the BR 18 × BRS 179 population. The presence of the BR 18 allele within the 7A region was consistently associated with lowered TGW in all trials, suggesting that these are the same QTL represented in both populations. DON accumulation QTL co-located with both the TGW QTL on chromosome 7A, with the BR 18 allele conferring reduced resistance to DON accumulation, however these associations were environmentally dependent. QTL associated with DON or FDK have been identified in the centromeric region of 7A in previous studies, with resistance being derived from varying sources [43, 58, 77]. However, there is a paucity of reported QTL associated with DON, TGW or FDK on the long arm of chromosome 7A. The relationship between TGW and DON is unclear both in this study, with weak positive and negative correlations being observed, and in the literature [65, 77–79], which may be due to the considerable environmental effect on DON accumulation.

In addition to the TGW QTL on chromosome 7A, a major TGW QTL on chromosome 6B was also stable across trial years in the BR 18 × BRS 179 population. The QTL *QME*.*Tgw*.*jic-6B* (16.8% variance) maps to the short arm of chromosome 6B (97.82–164.39 Mb), with the BRS 179 allele providing low TGW at this locus. The gene *TaGW2-6B*, which is associated with grain weight in bread wheat, is also present on chromosome 6B at 291.76 Mb [80] but sits outside the QTL interval identified for *QME*.*Tgw*.*jic-6B*. The major FHB resistance gene *Fhb2* has been mapped to the short arm of 6B between the markers *gwm644* and *gwm133*, with reduced FDK and FHB severity being derived from the cultivar Sumai 3 [81]. FDK has been previously mapped on the short arm of chromosome 6B within the *Fhb2* region in Brazilian germplasm, using bi-parental mapping with the cultivar Frontana [23] and through genome wide association mapping using a panel of wheat breeding lines and varieties relevant to Brazilian wheat breeding [18]. The *QME*.*Tgw*.*jic-6B* interval sits within 10.18 Mb of *Kukri_c25377_240*, the closest significant marker associated with FDK in the study by Mellers et al. [18], and is 53.57 Mb outside the *Fhb2* fine mapped region [81, 82]. In our study, *QME*.*Tgw*.*jic-6B* was not associated with other FHB traits, such as FHB severity and DON accumulation, as reported in other studies [23, 81], which may indicate that this QTL is not due to the presence of *Fhb2*. Considering the effect of this QTL on TGW, further investigation to confirm the presence/absence of *Fhb2* within the cultivars used in this study may be worthwhile.

FHB causes bleaching of infected wheat heads prior to senescence, a symptom which is also characteristic of wheat blast, caused by *M*. *oryzae* MoT pathotype [25]. Both pathogens are hemibiotrophic, but while resistance to blast is isolate-specific and primarily governed by major resistance (*R*) genes [83], FHB resistance is quantitative and race non-specific. The cultivar Sumai 3 displays high levels of FHB resistance, yet is susceptible to blast disease [27]. Contrastingly, the cultivar Milan is resistant to blast, but susceptible to FHB. However, it has been unclear whether this resistance differential is due to a lack of common resistance genes which confer resistance to both pathogens, or due to pleiotropy or linkage associated with specific resistances. Trade-off in resistance effects have been reported previously. For example, wheat TILLING (targeted induced local lesions in genomes) lines possessing the loss-of-function *Ta-mlo* powdery mildew resistance alleles show increased susceptibility to *M*. *oryzae* MoT pathotypes [84]. The resistance associated genes peroxidase (*Pox2*) and cinnamoyl-CoA reductase (*CRR*), which have roles in cell wall lignification, are differentially upregulated following infection with FHB and blast [27], suggesting there may be specific interactions between the two pathogens and the wheat host. BR 18 displays consistent wheat blast resistance [85], whilst BRS 179 is moderately resistant/susceptible and Anahuac 75 is highly susceptible [29, 86]. We previously identified genetic loci associated with blast resistance on chromosomes 1A, 2B, 4A, 4B, 5A and 6A in the Anahuac 75 × BR 18 and BR 18 × BRS 179 populations [30], with resistance at the seedling and head stage being governed by different genomic regions. In the present study, BRS 179 displayed the greatest FHB resistance whereas BR 18 and Anahuac 75 were moderately and highly susceptible to FHB, respectively. However, none of the QTL associated with FHB resistance associated traits mapped to the same location as those we previously associated with wheat blast. This suggests that whilst the cultivars in this study display contrasting disease responses to FHB and blast, this is not due to a trade-off caused by pleiotropy or linkage with genes for resistance to these two diseases and that separate pathways may mediate resistance to each pathogen. While providing evidence that selecting for resistance to FHB will not compromise resistance to wheat blast, it does demonstrate that breeders will need to combine resistances to the two diseases separately using methods such as phenotypic selection, marker-assisted selection (MAS) or genomic prediction. This should allow the generation of resistant cultivars suitable for cultivation in regions of the world, such as Brazil, where both FHB and blast pose a serious threat to wheat production.

## Conclusions

In this study, we aimed to determine the genetic basis of FHB resistance in Brazilian wheat cultivars that lack major resistance genes, such as *Fhb1*. Several QTL were identified that were associated with FHB related traits, demonstrating that resistance is conferred by multiple loci and not a single, alternative major-effect gene. We identified a major, novel QTL associated with reduced DON accumulation, *QME*.*Don*.*jic-4B*, on the long arm of chromosome 4B, and a major QTL, *QME*.*Tgw*.*jic-6B*, associated with TGW on the short arm of chromosome 6B. Given the increasing frequency of severe FHB outbreaks worldwide, and therefore the greater mycotoxin risks associated with FHB infected grain, these QTL could prove to be useful breeding targets to improve grain quality in Brazil and other countries affected by FHB. By using MAS or other approaches to pyramid these QTL with major genes, such as *Fhb1*, it is possible that greater resistance could be achieved, therefore reducing the reliance on fungicides and providing a reduced risk of crop losses due to FHB.

## Supporting information

S1 Fig QTL identified on 1D in the Anahuac 75 × BR 18 F_6_ RIL population. (PPTX)

S2 Fig QTL identified on 2A in the Anahuac 75 × BR 18 F_6_ RIL population. (PPTX)

S3 Fig QTL identified on 2D in the Anahuac 75 × BR 18 F_6_ RIL population. (PPTX)

S4 Fig QTL identified on 3B in the Anahuac 75 × BR 18 F_6_ RIL population. (PPTX)

S5 Fig QTL identified on 4B in the Anahuac 75 × BR 18 F_6_ RIL population. (PPTX)

S6 Fig QTL identified on 4D in the Anahuac 75 × BR 18 F_6_ RIL population. (PPTX)

S7 Fig QTL identified on 5B in the Anahuac 75 × BR 18 F_6_ RIL population. (PPTX)

S8 Fig QTL identified on 7A in the Anahuac 75 × BR 18 F_6_ RIL population. (PPTX)

S9 Fig QTL identified on 1A in the BR 18 × BRS 179 F_6_ RIL population. (PPTX)

S10 Fig QTL identified on 2A in the BR 18 × BRS 179 F_6_ RIL population. (PPTX)

S11 Fig QTL identified on 2B in the BR 18 × BRS 179 F_6_ RIL population. (PPTX)

S12 Fig QTL identified on 2D in the BR 18 × BRS 179 F_6_ RIL population. (PPTX)

S13 Fig QTL identified on 3B in the BR 18 × BRS 179 F_6_ RIL population. (PPTX)

S14 Fig QTL identified on 4A in the BR 18 × BRS 179 F_6_ RIL population. (PPTX)

S15 Fig QTL identified on 4D in the BR 18 × BRS 179 F_6_ RIL population. (PPTX)

S16 Fig QTL identified on 6A in the BR 18 × BRS 179 F_6_ RIL population. (PPTX)

S17 Fig QTL identified on 6B in the BR 18 × BRS 179 F_6_ RIL population. (PPTX)

S18 Fig QTL identified on 7A in the BR 18 × BRS 179 F_6_ RIL population. (PPTX)

S1 File Phenotypic data for the Anahuac 75 × BR 18 RIL population and the BR 18 × BRS 179 RIL population. (XLSX)
