# Supplementary figures and images for: Identification of Fusarium head blight resistance loci in two Brazilian wheat mapping populations
Source: PLoS One. 2021 Mar 8;16(3):e0248184. doi: 10.1371/journal.pone.0248184 (PMC7939358; doi:10.1371/journal.pone.0248184)

## Slide 1
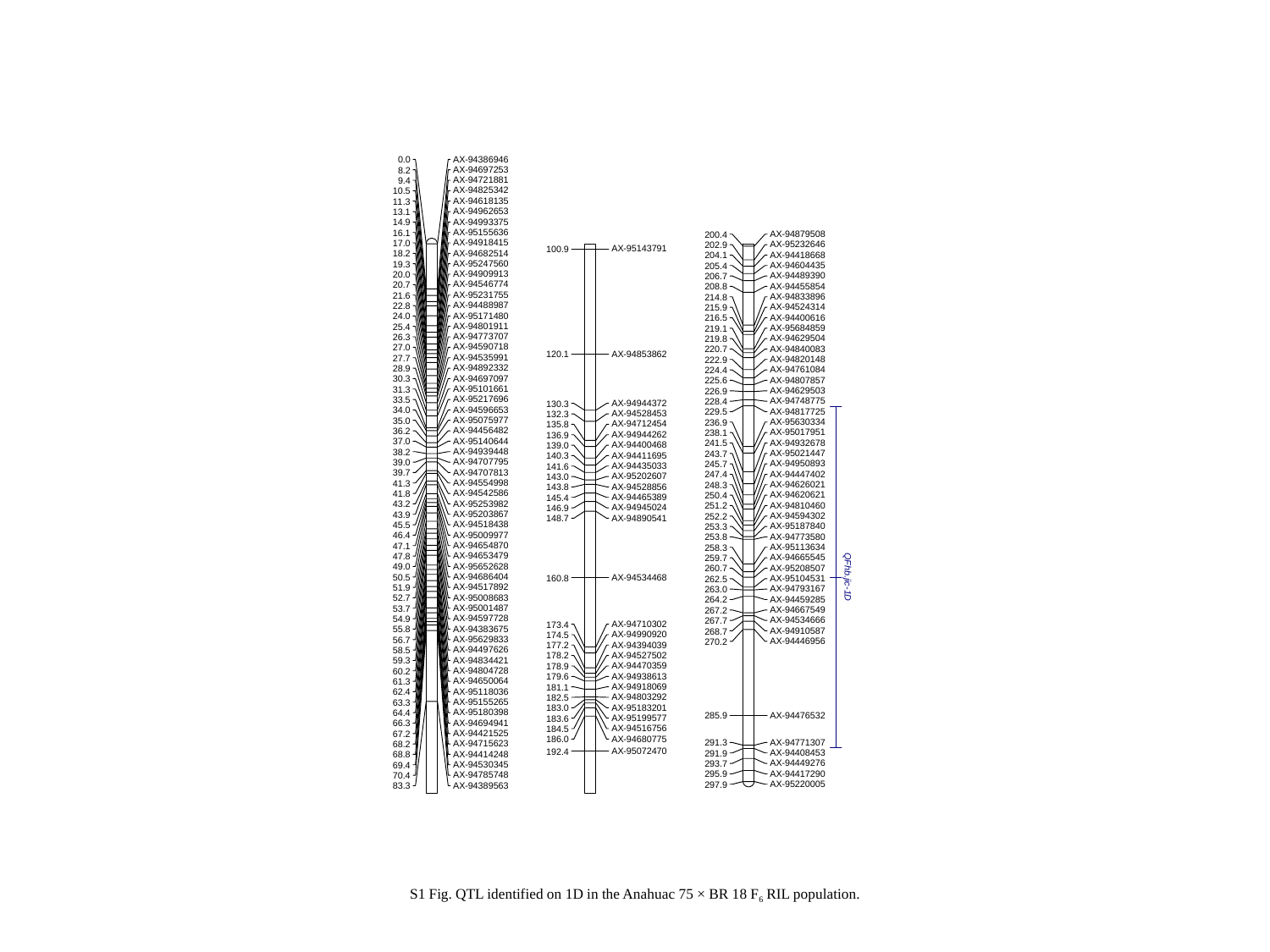

S1 Fig. QTL identified on 1D in the Anahuac 75 × BR 18 F6 RIL population.

Supplement: S1 Fig — (PPTX) [file pone.0248184.s001.pptx]

## Slide 1
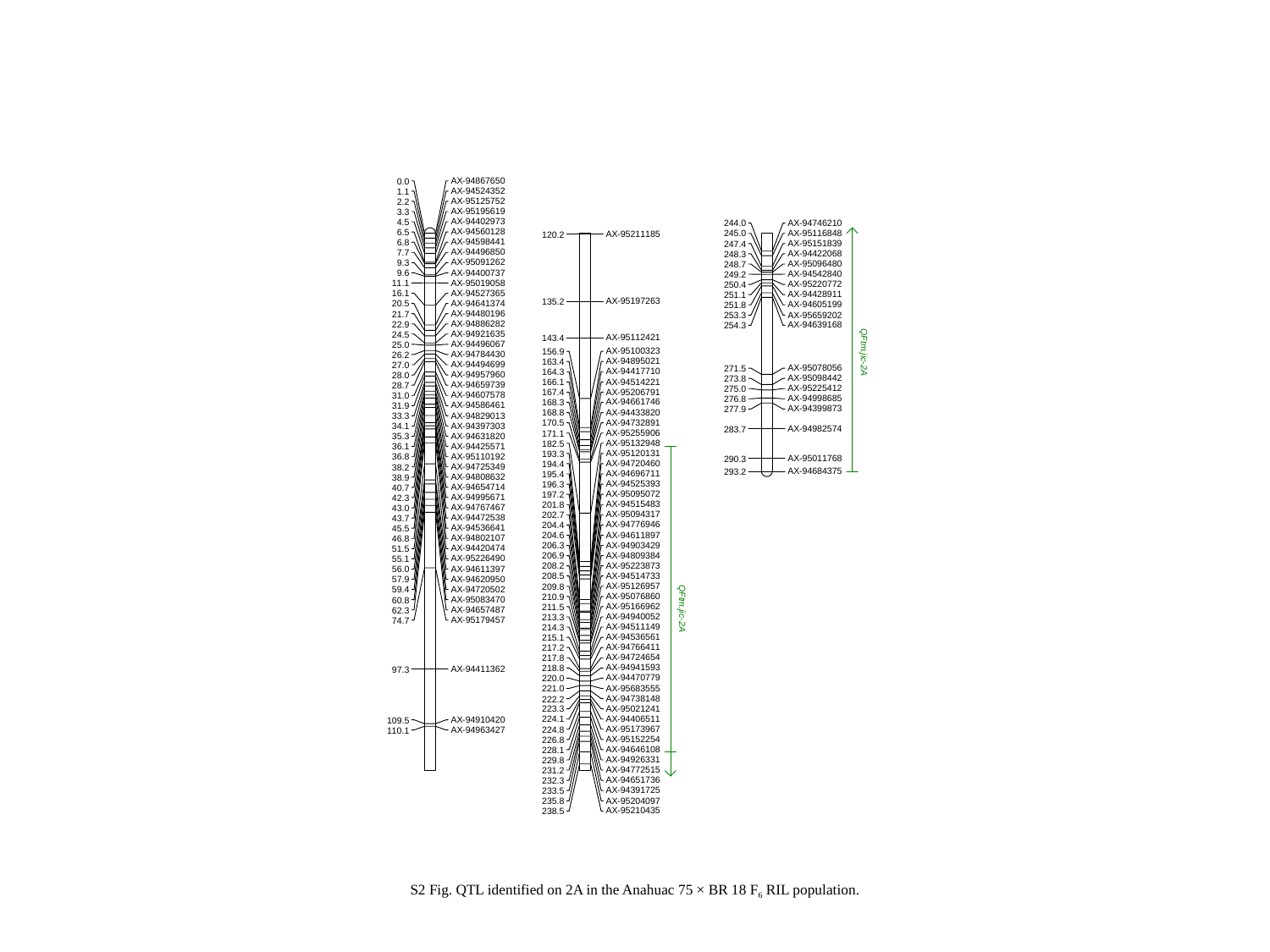

S2 Fig. QTL identified on 2A in the Anahuac 75 × BR 18 F6 RIL population.

Supplement: S2 Fig — (PPTX) [file pone.0248184.s002.pptx]

## Slide 1
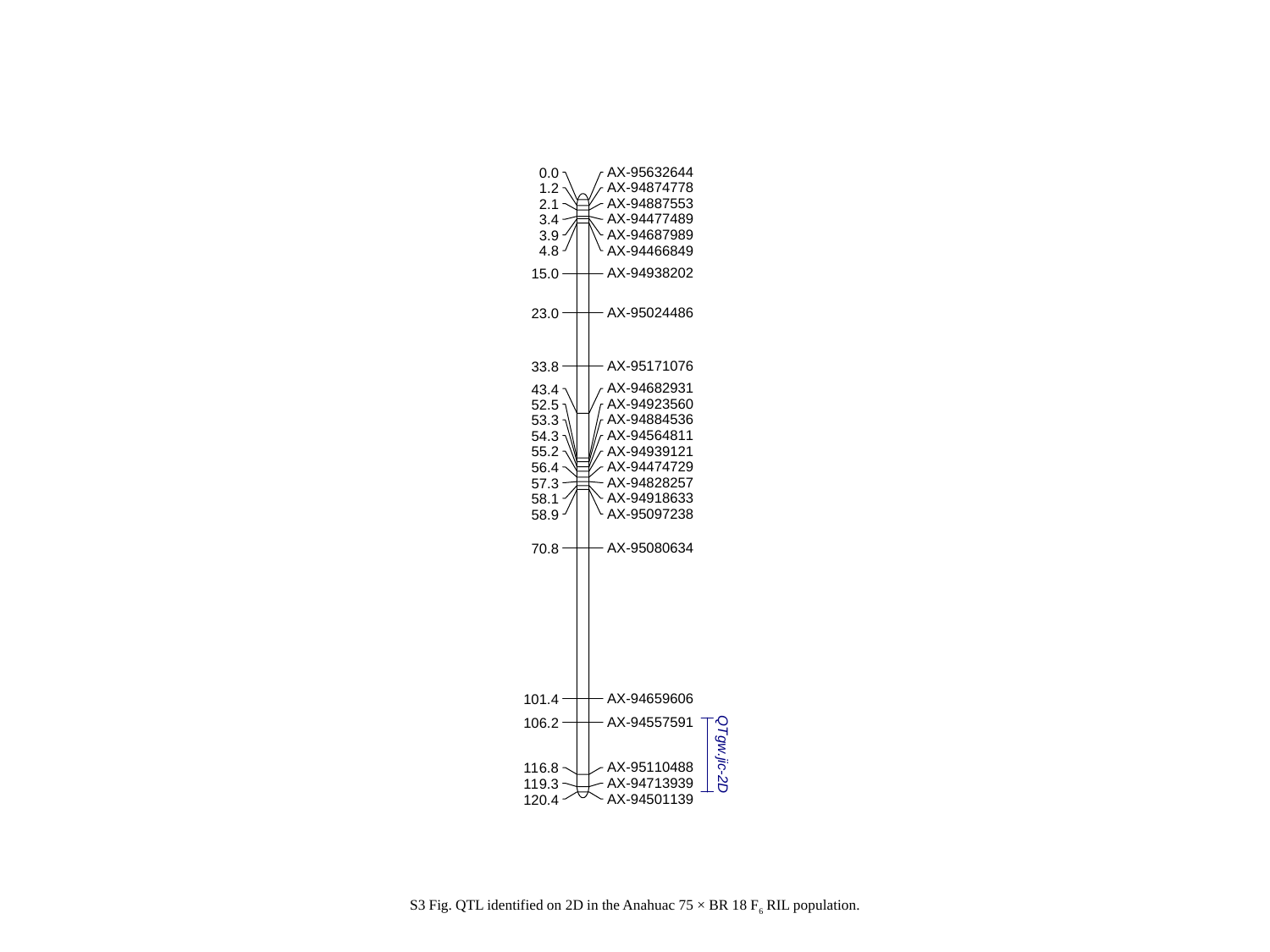

S3 Fig. QTL identified on 2D in the Anahuac 75 × BR 18 F6 RIL population.

Supplement: S3 Fig — (PPTX) [file pone.0248184.s003.pptx]

## Slide 1
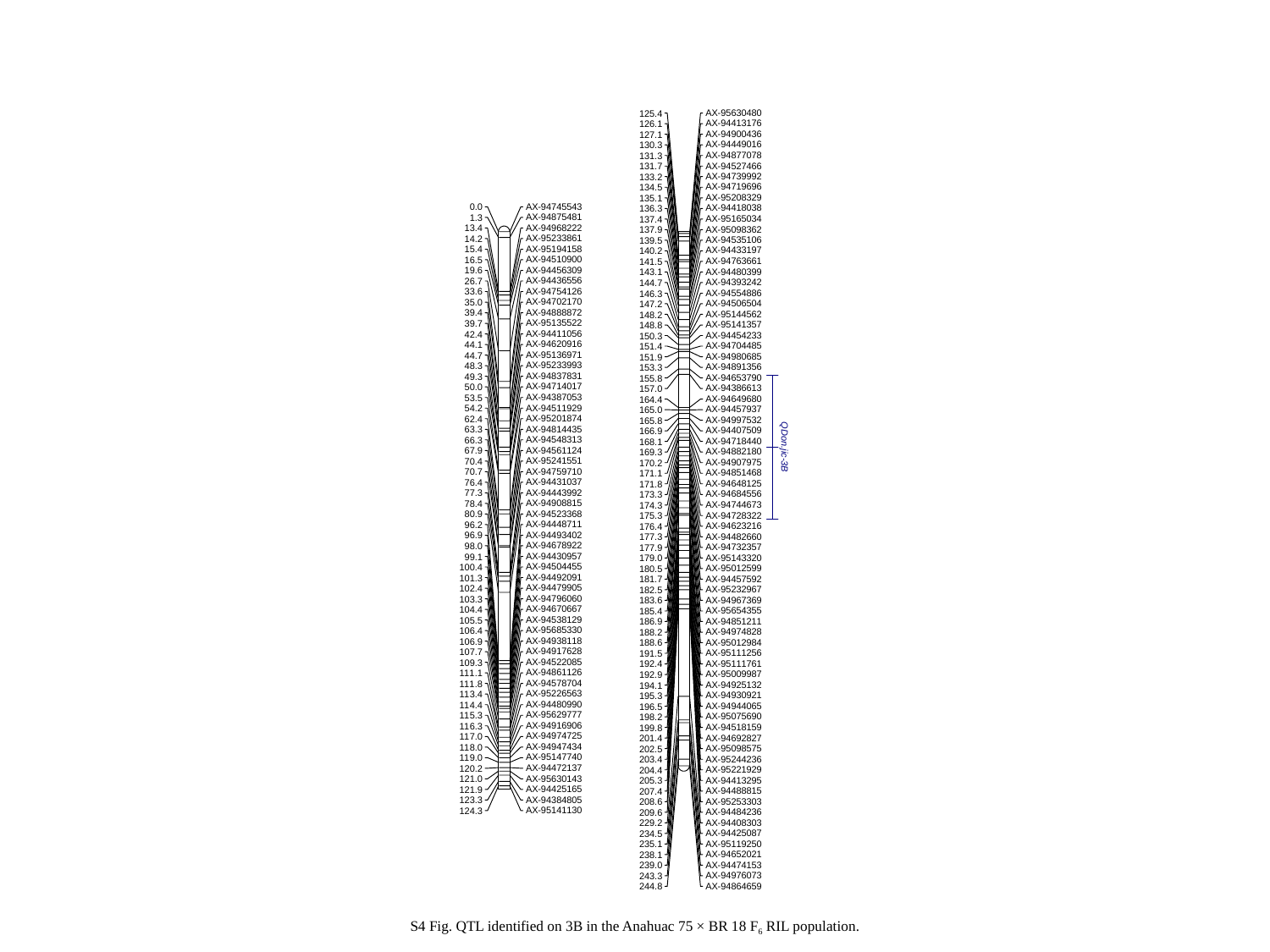

S4 Fig. QTL identified on 3B in the Anahuac 75 × BR 18 F6 RIL population.

Supplement: S4 Fig — (PPTX) [file pone.0248184.s004.pptx]

## Slide 1
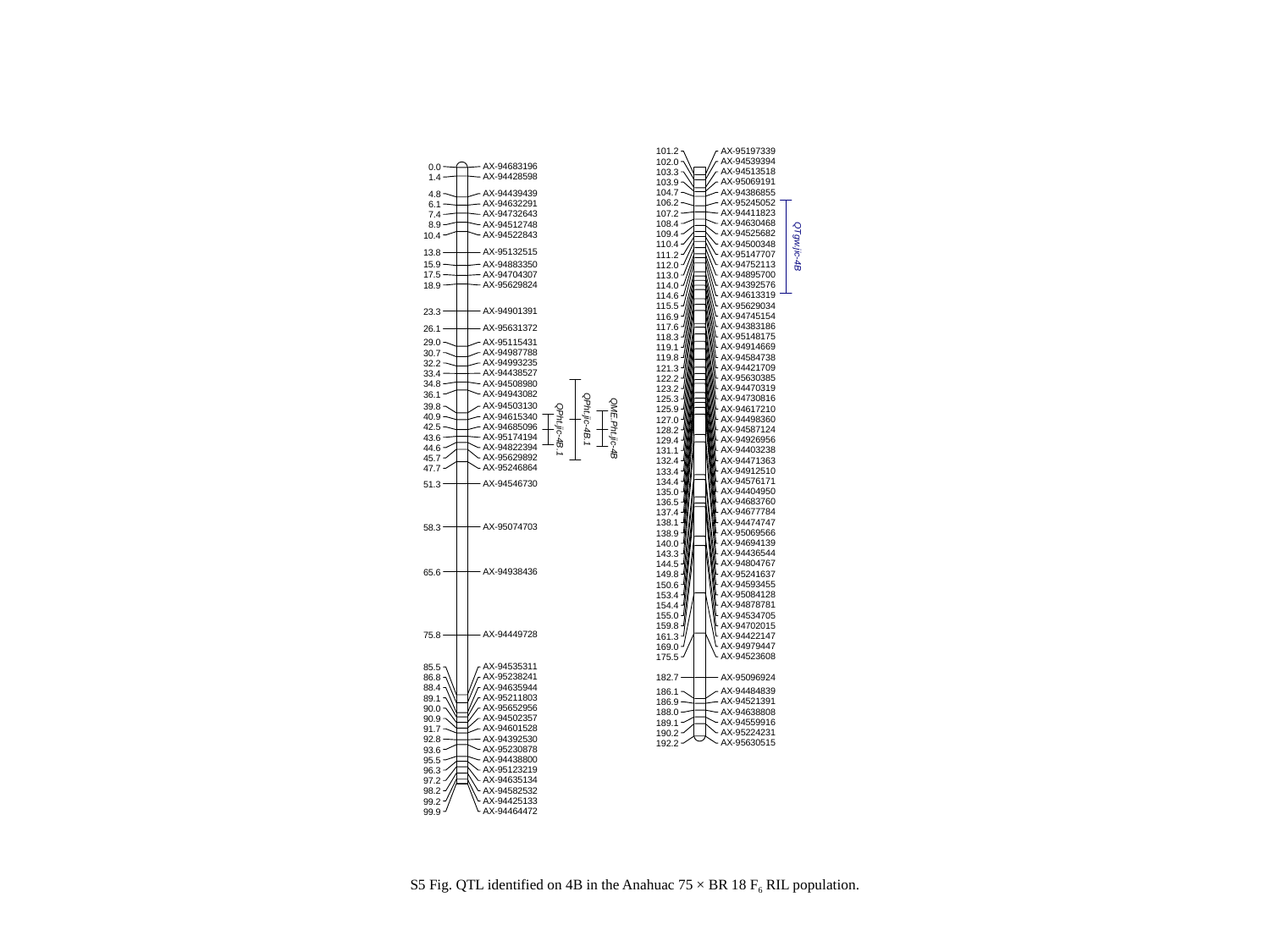

S5 Fig. QTL identified on 4B in the Anahuac 75 × BR 18 F6 RIL population.

Supplement: S5 Fig — (PPTX) [file pone.0248184.s005.pptx]

## Slide 1
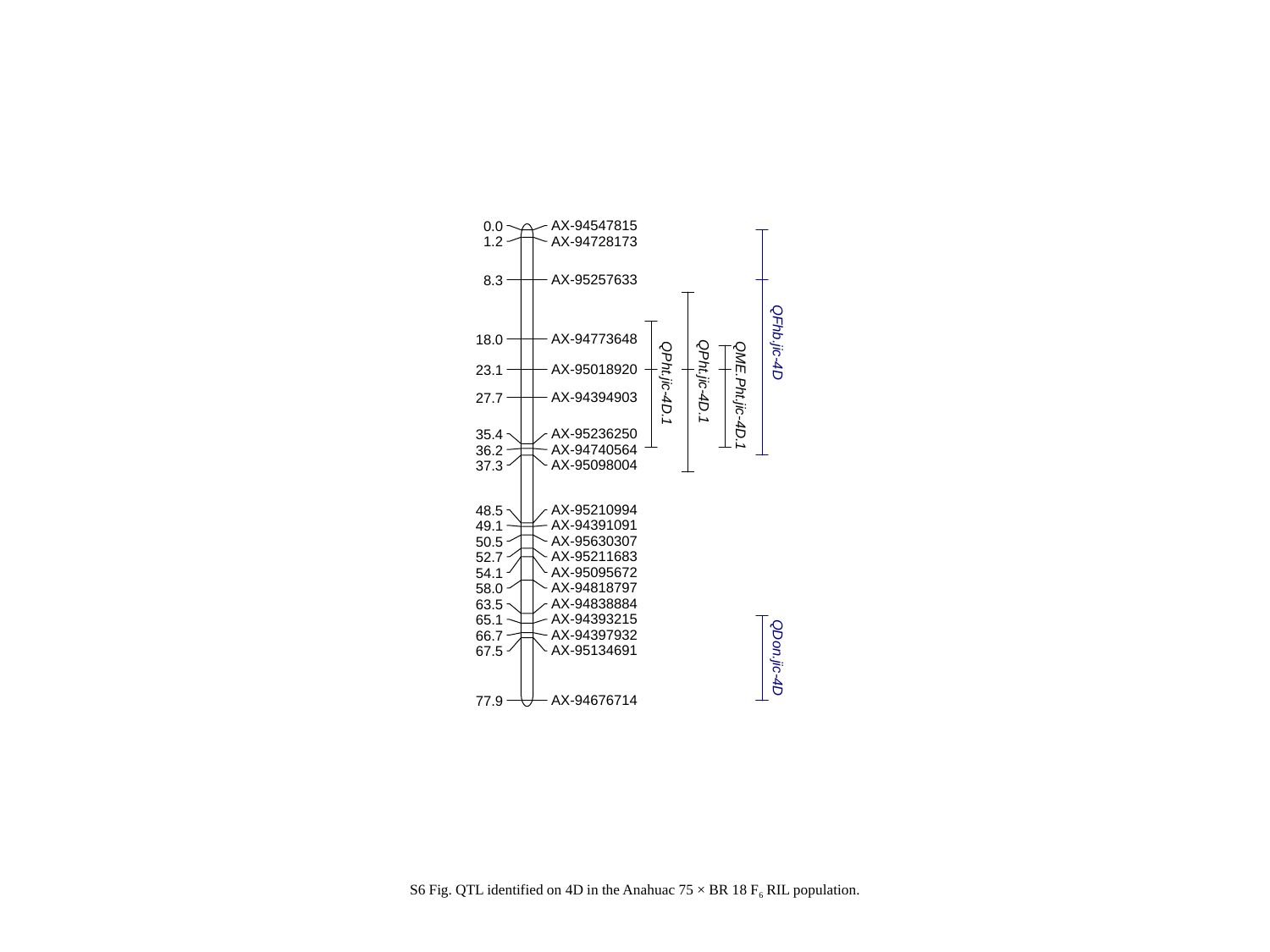

S6 Fig. QTL identified on 4D in the Anahuac 75 × BR 18 F6 RIL population.

Supplement: S6 Fig — (PPTX) [file pone.0248184.s006.pptx]

## Slide 1
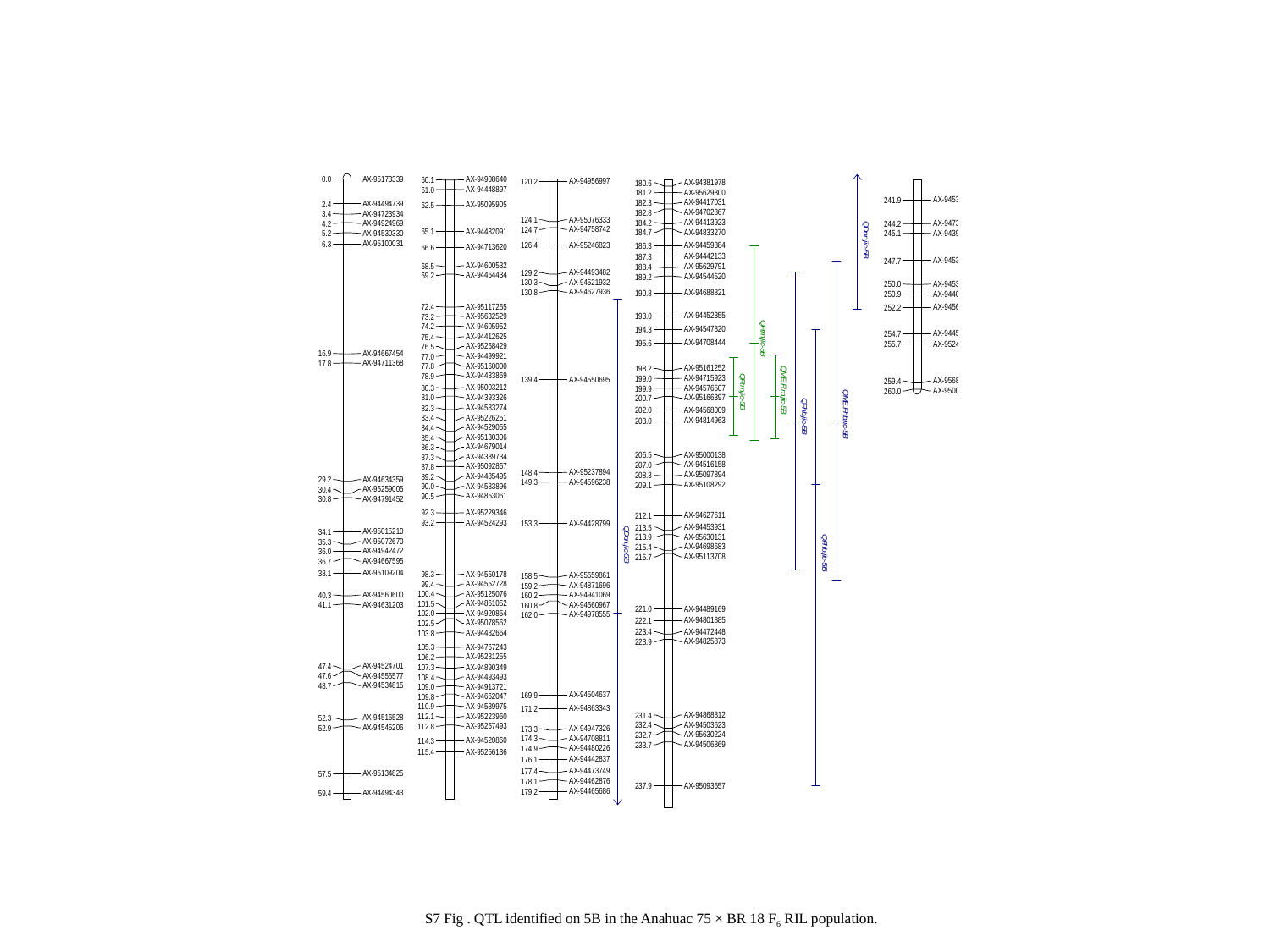

S7 Fig . QTL identified on 5B in the Anahuac 75 × BR 18 F6 RIL population.

Supplement: S7 Fig — (PPTX) [file pone.0248184.s007.pptx]

## Slide 1
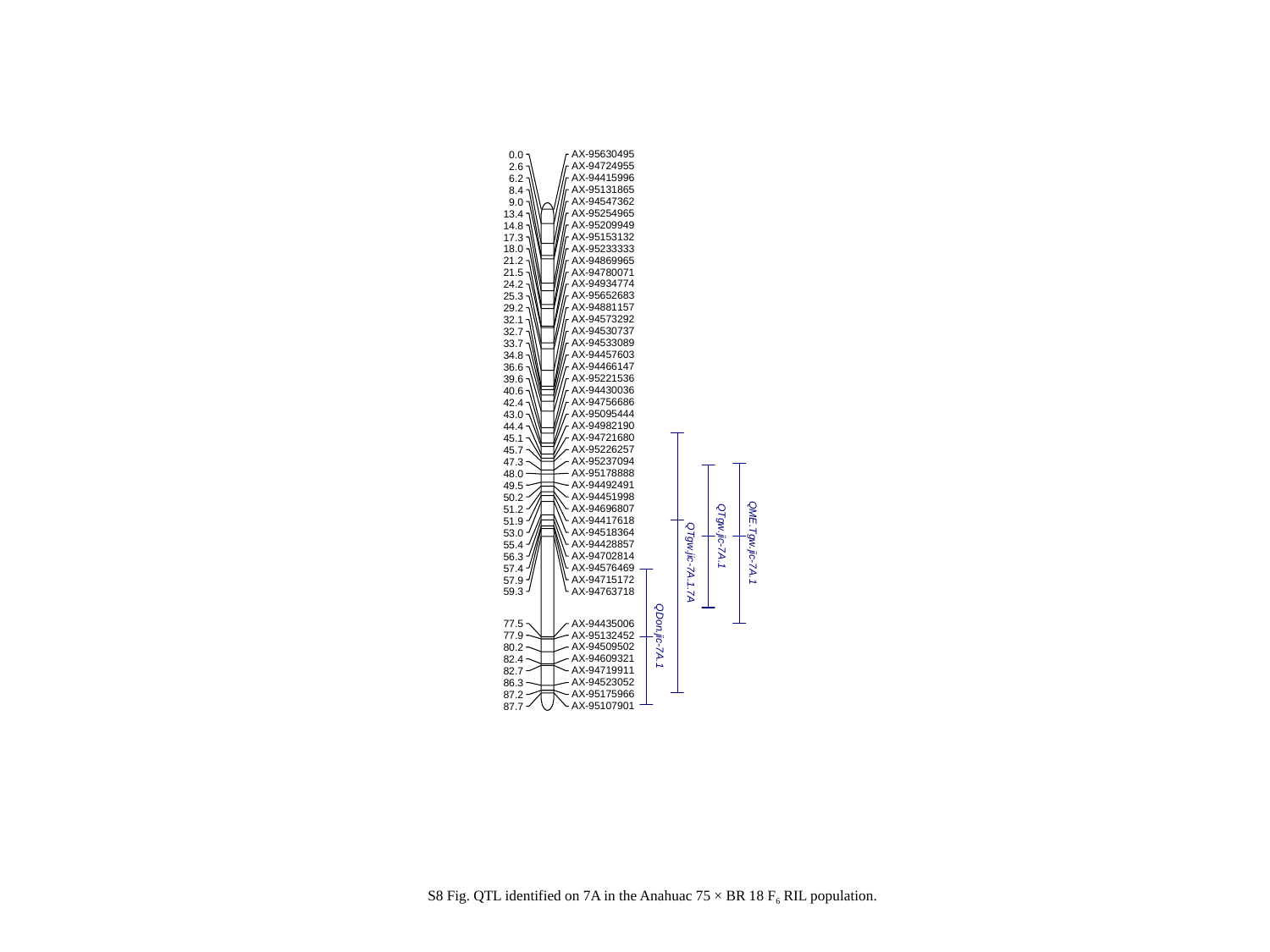

S8 Fig. QTL identified on 7A in the Anahuac 75 × BR 18 F6 RIL population.

Supplement: S8 Fig — (PPTX) [file pone.0248184.s008.pptx]

## Slide 1
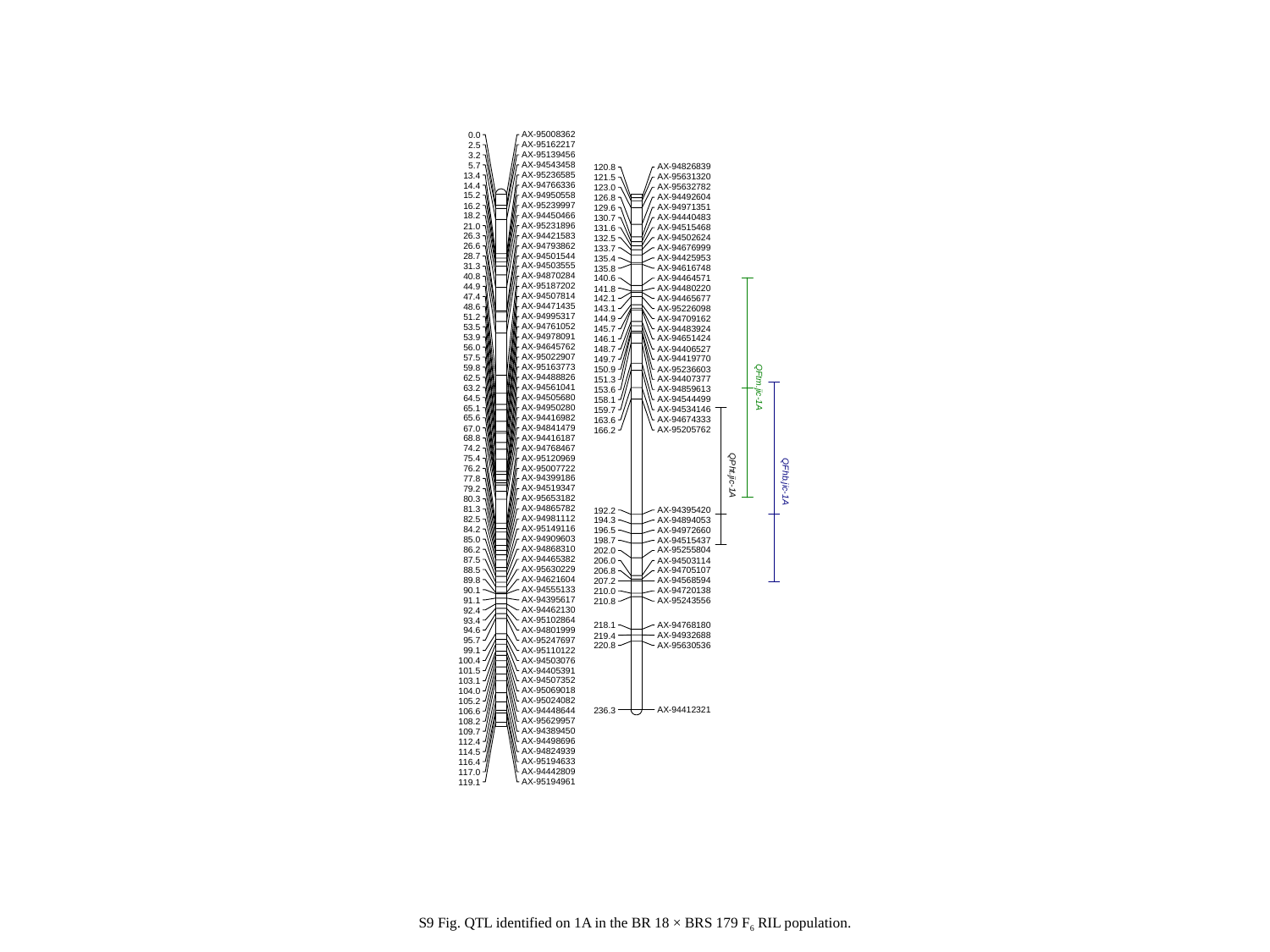

S9 Fig. QTL identified on 1A in the BR 18 × BRS 179 F6 RIL population.

Supplement: S9 Fig — (PPTX) [file pone.0248184.s009.pptx]

## Slide 1
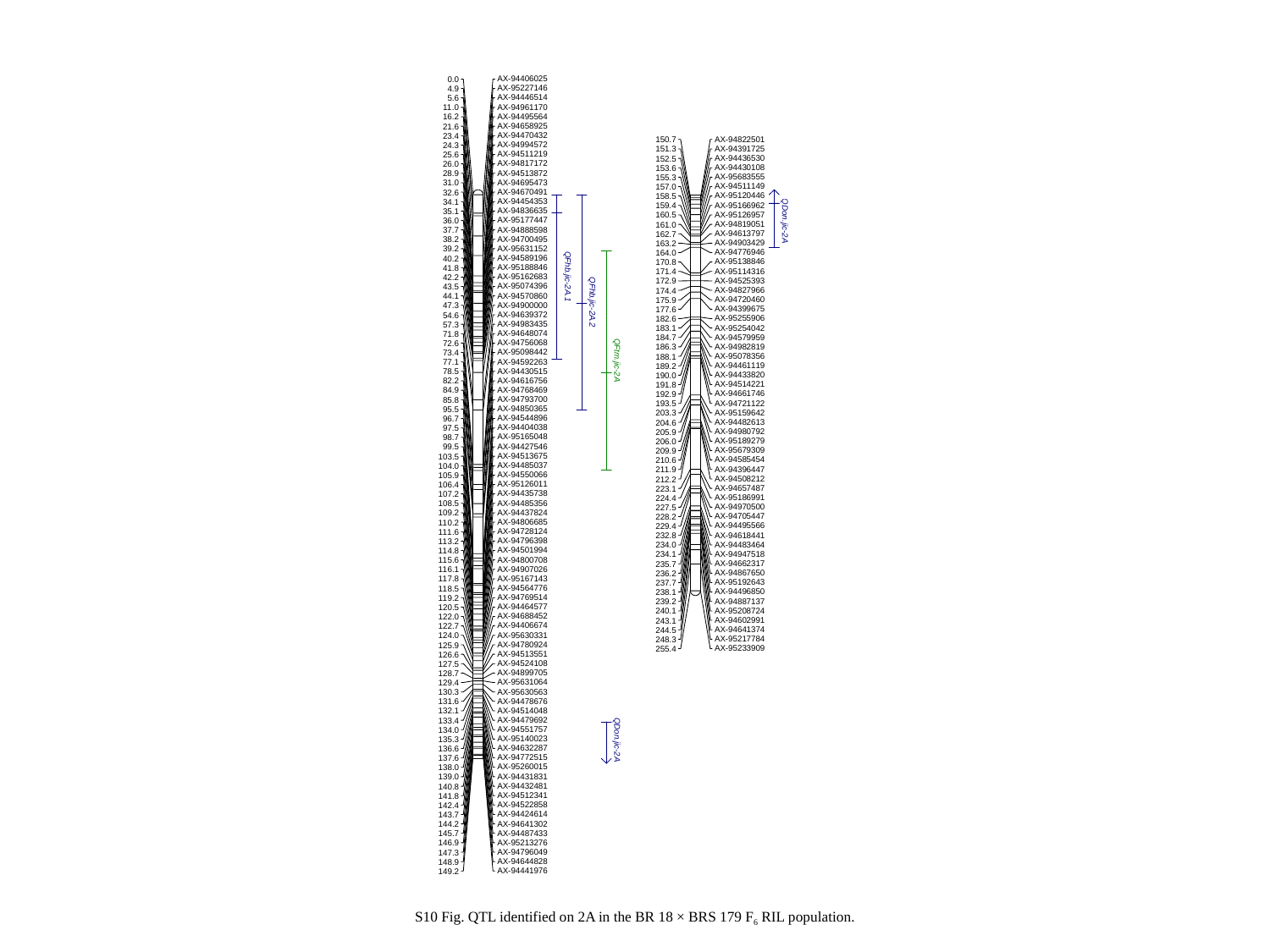

S10 Fig. QTL identified on 2A in the BR 18 × BRS 179 F6 RIL population.

Supplement: S10 Fig — (PPTX) [file pone.0248184.s010.pptx]

## Slide 1
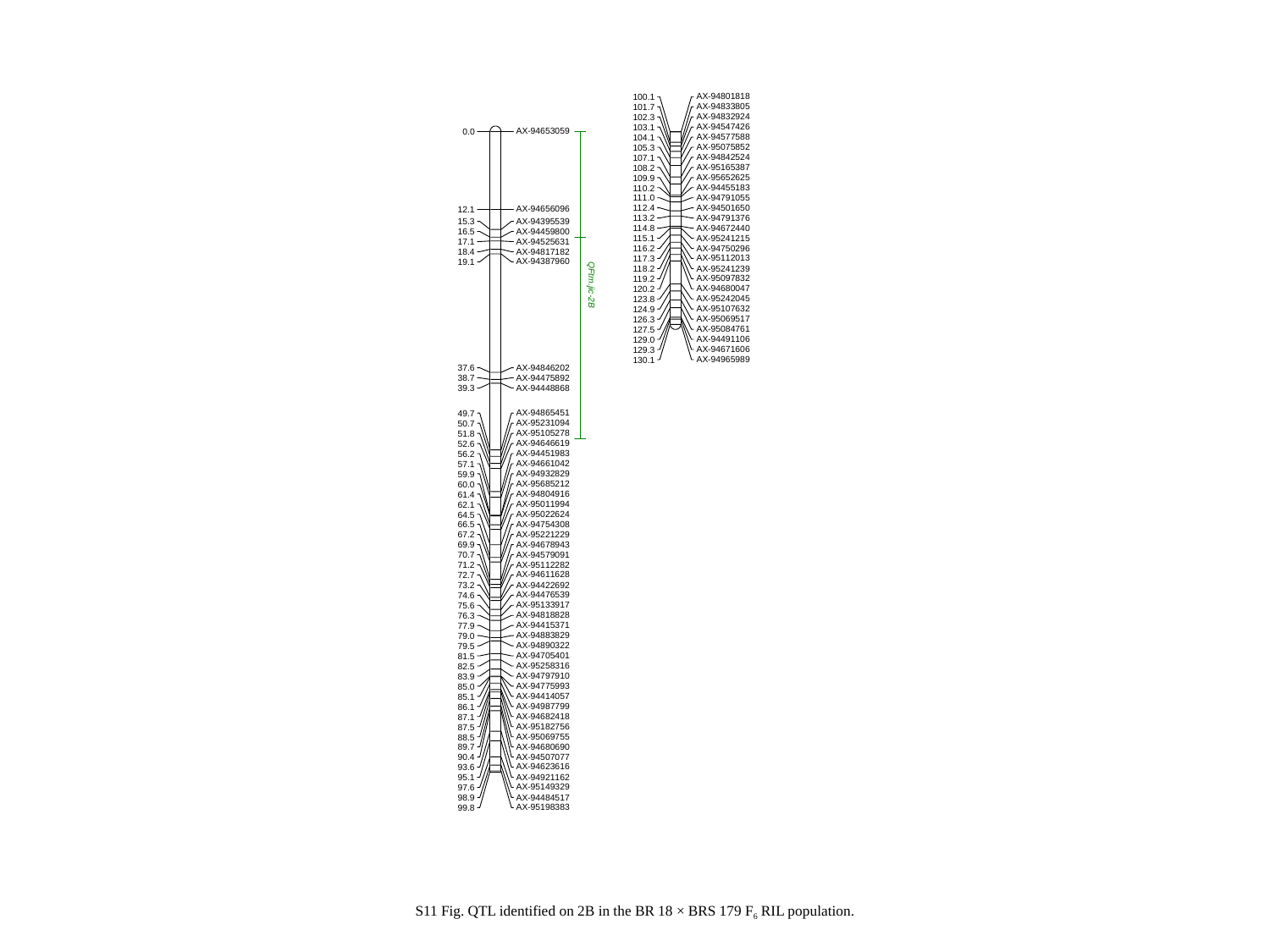

S11 Fig. QTL identified on 2B in the BR 18 × BRS 179 F6 RIL population.

Supplement: S11 Fig — (PPTX) [file pone.0248184.s011.pptx]

## Slide 1
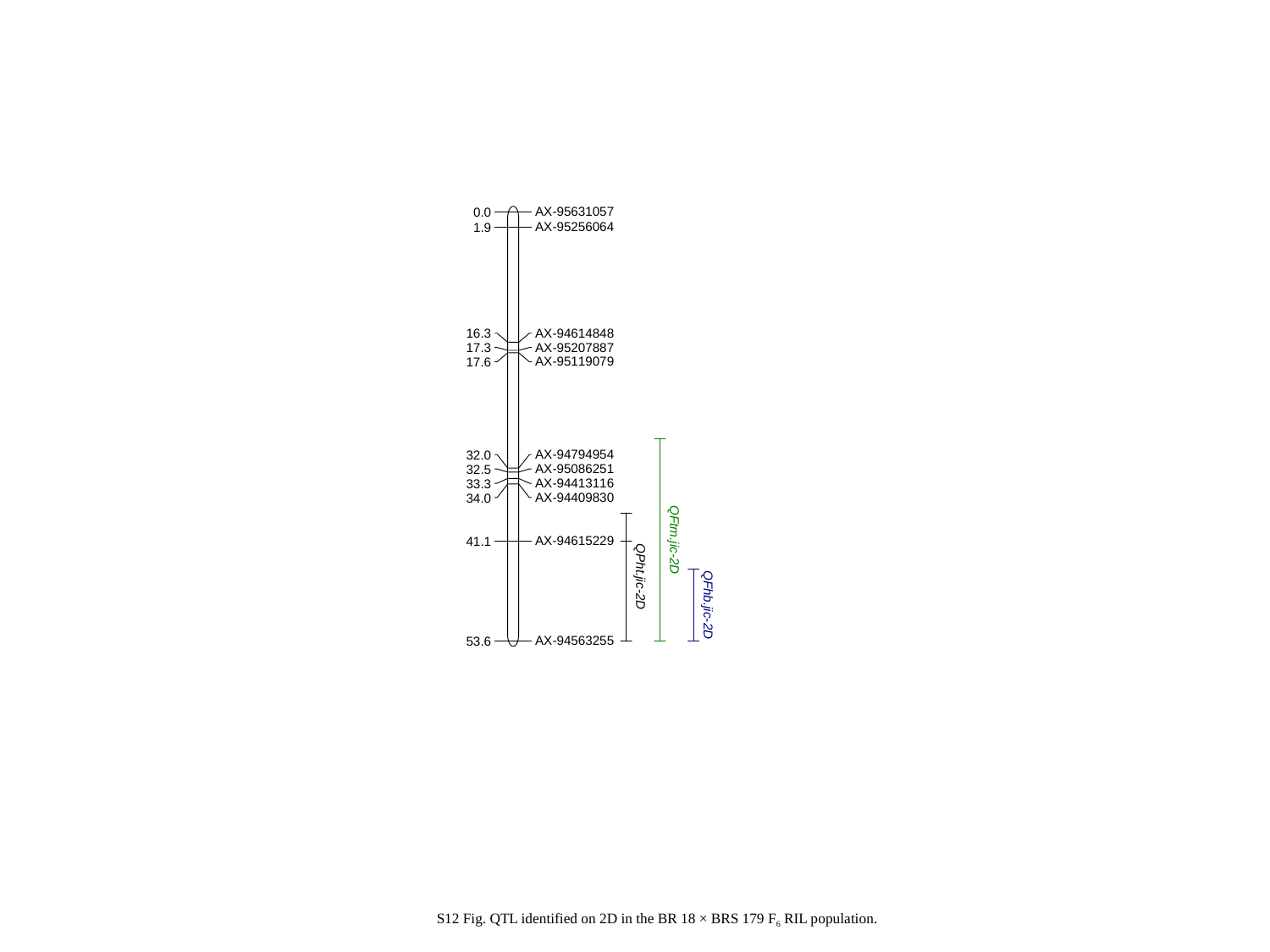

S12 Fig. QTL identified on 2D in the BR 18 × BRS 179 F6 RIL population.

Supplement: S12 Fig — (PPTX) [file pone.0248184.s012.pptx]

## Slide 1
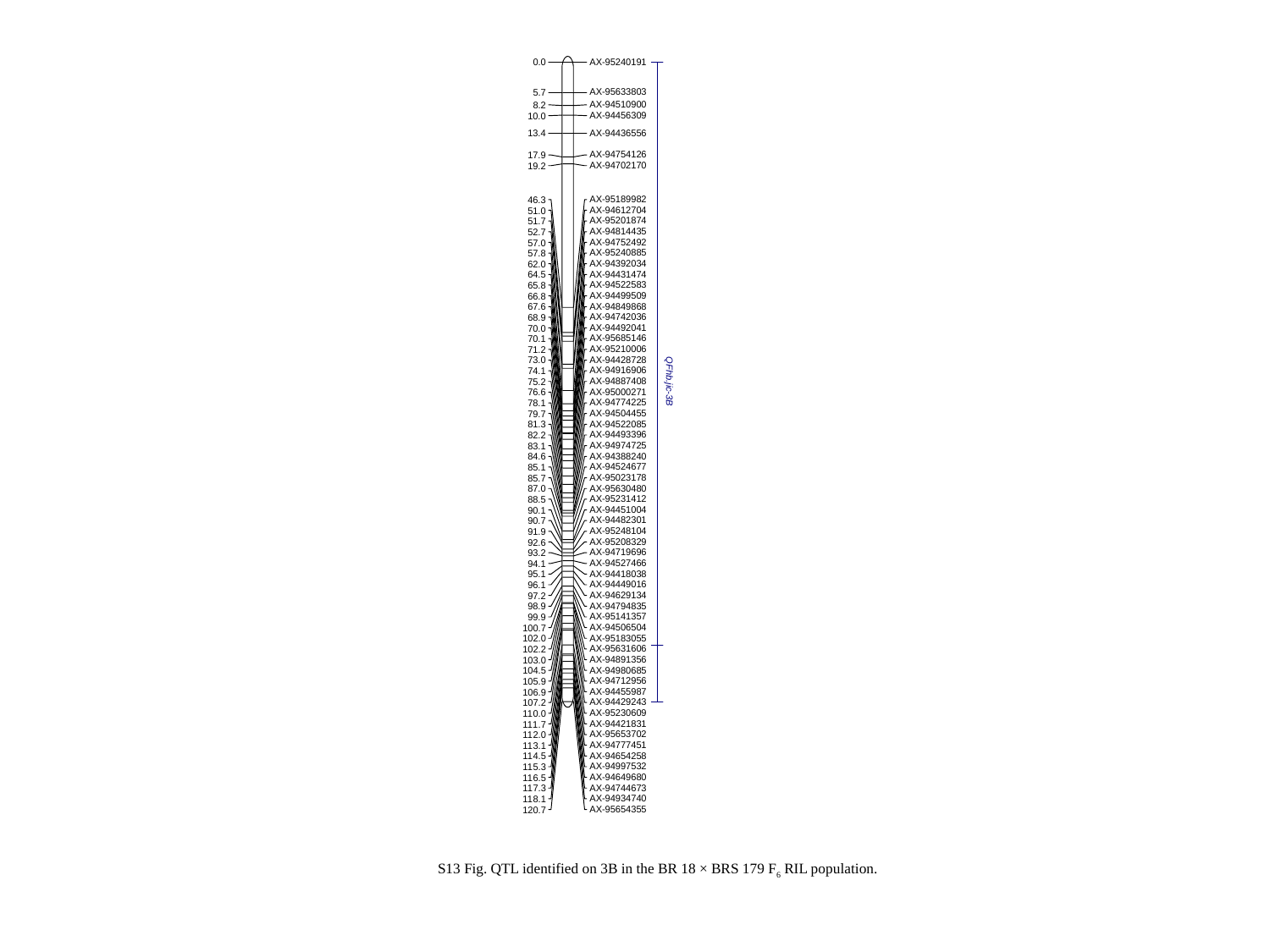

S13 Fig. QTL identified on 3B in the BR 18 × BRS 179 F6 RIL population.

Supplement: S13 Fig — (PPTX) [file pone.0248184.s013.pptx]

## Slide 1
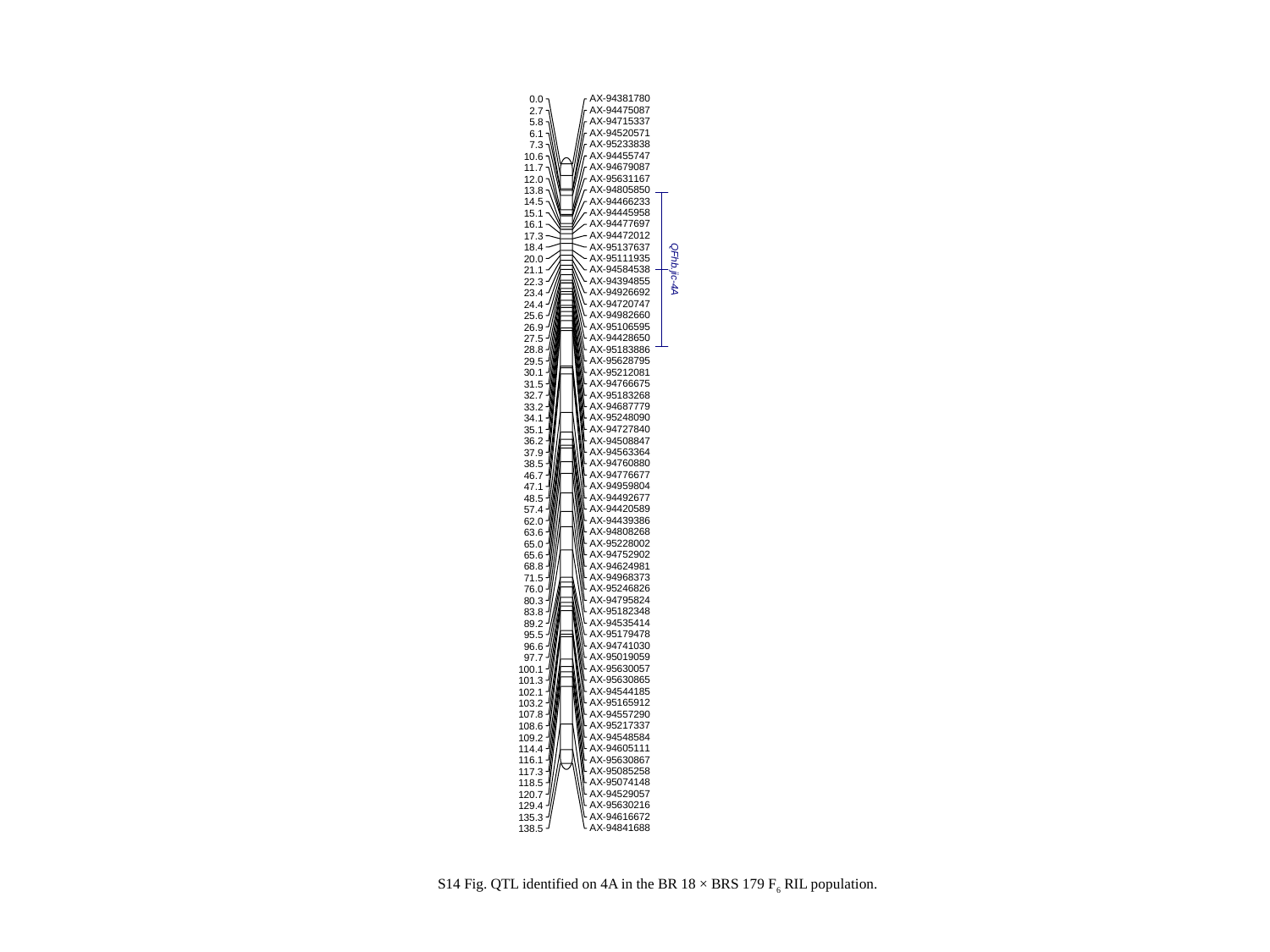

S14 Fig. QTL identified on 4A in the BR 18 × BRS 179 F6 RIL population.

Supplement: S14 Fig — (PPTX) [file pone.0248184.s014.pptx]

## Slide 1
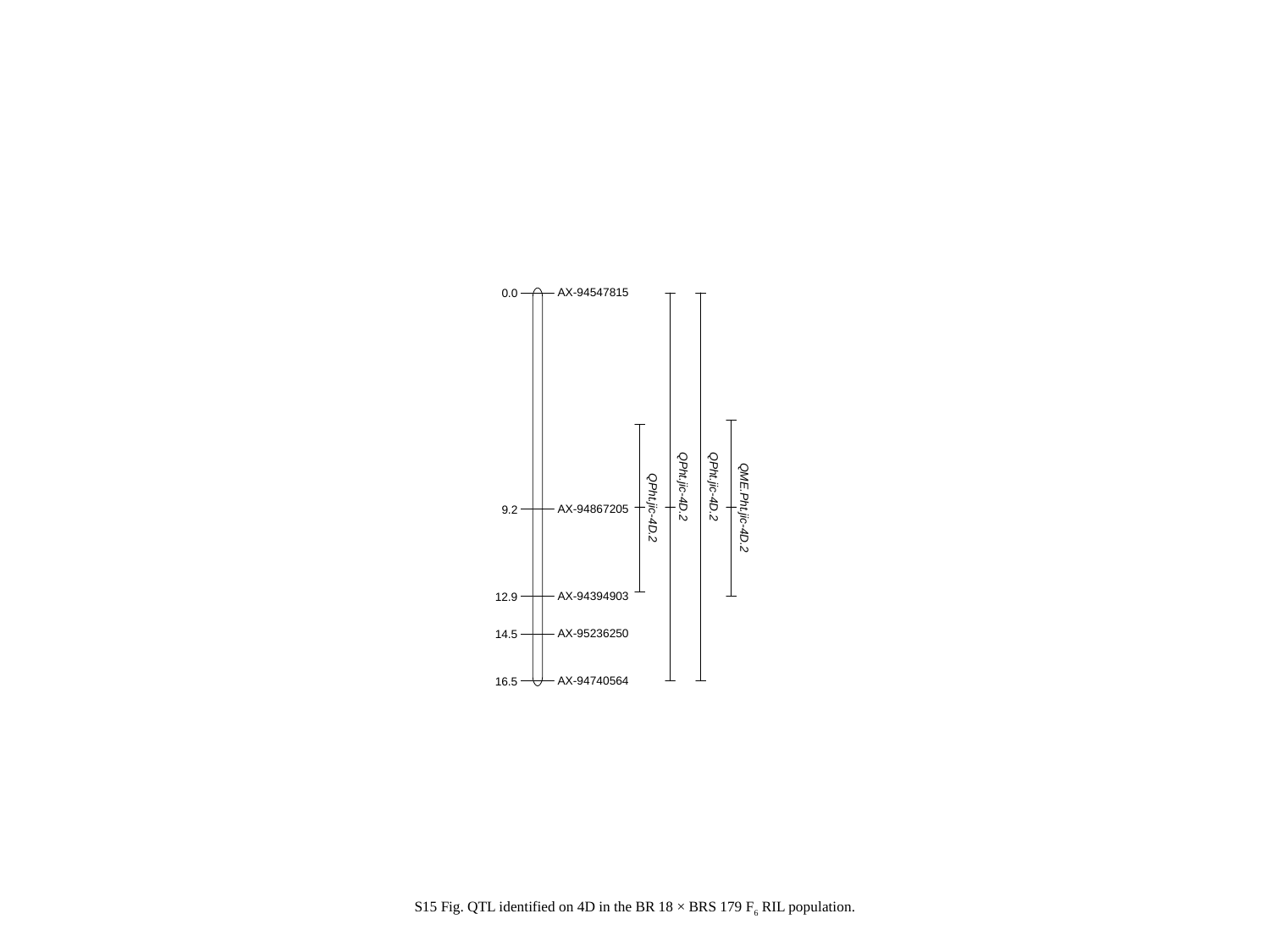

S15 Fig. QTL identified on 4D in the BR 18 × BRS 179 F6 RIL population.

Supplement: S15 Fig — (PPTX) [file pone.0248184.s015.pptx]

## Slide 1
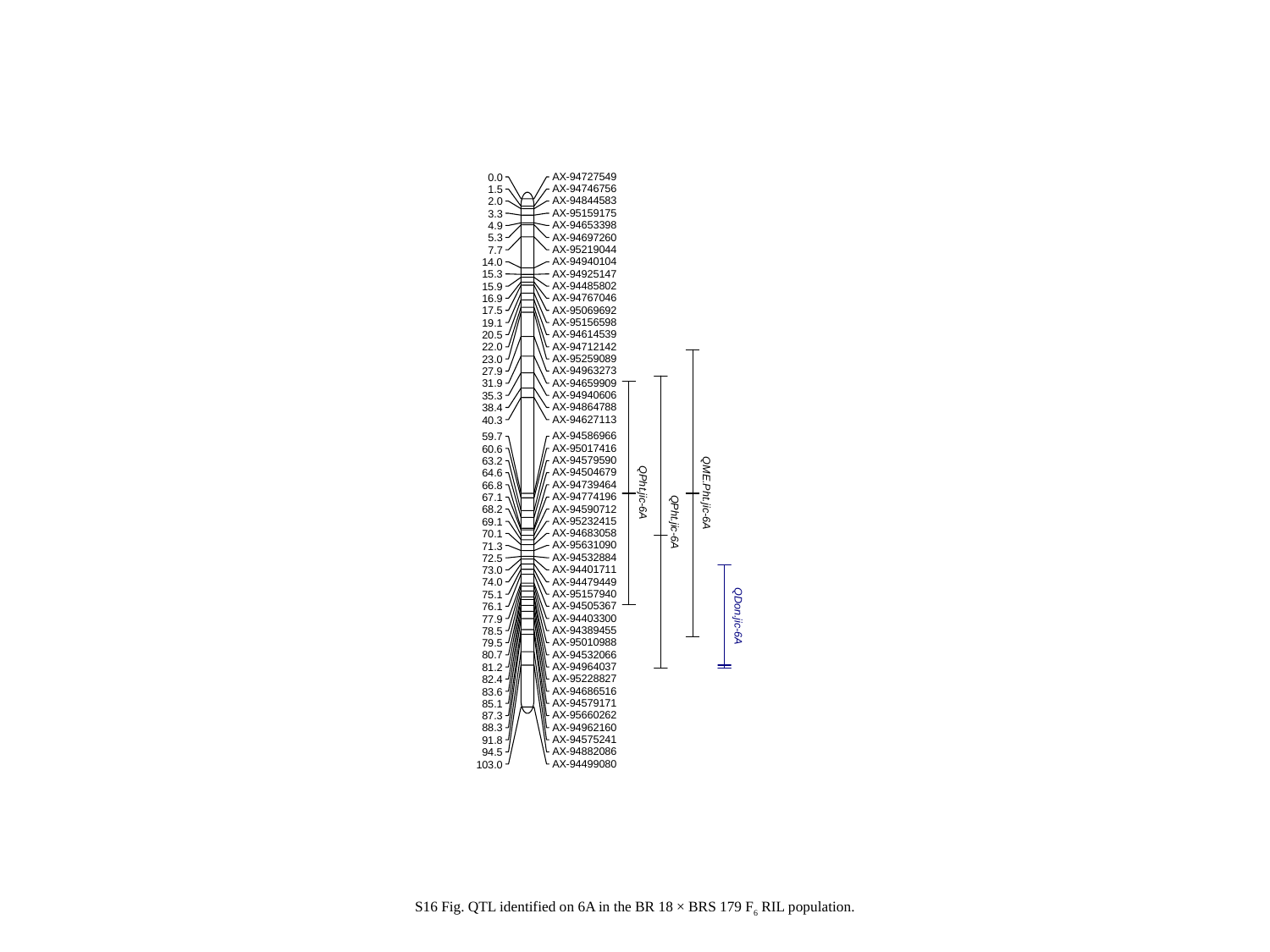

S16 Fig. QTL identified on 6A in the BR 18 × BRS 179 F6 RIL population.

Supplement: S16 Fig — (PPTX) [file pone.0248184.s016.pptx]

## Slide 1
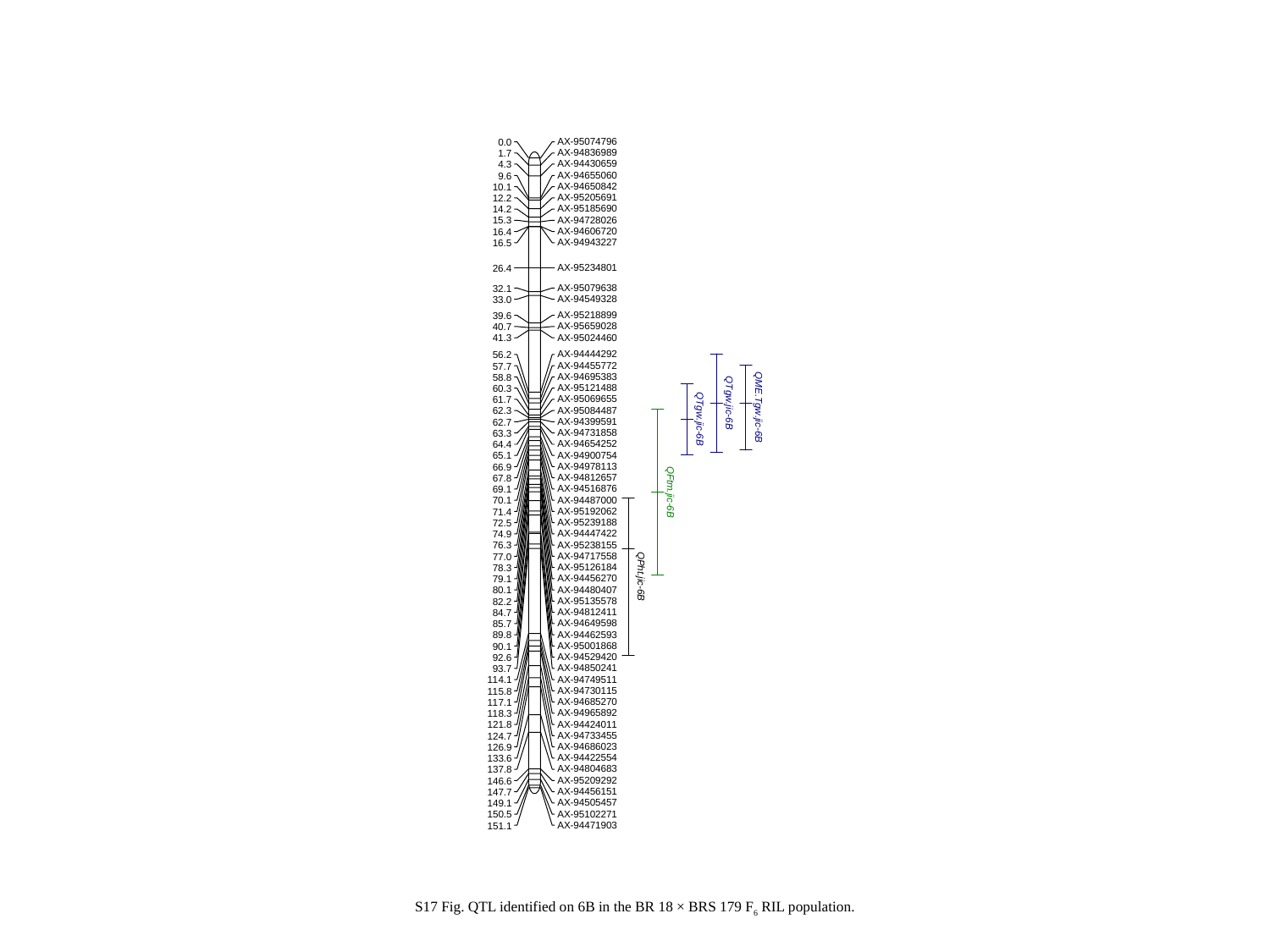

S17 Fig. QTL identified on 6B in the BR 18 × BRS 179 F6 RIL population.

Supplement: S17 Fig — (PPTX) [file pone.0248184.s017.pptx]

## Slide 1
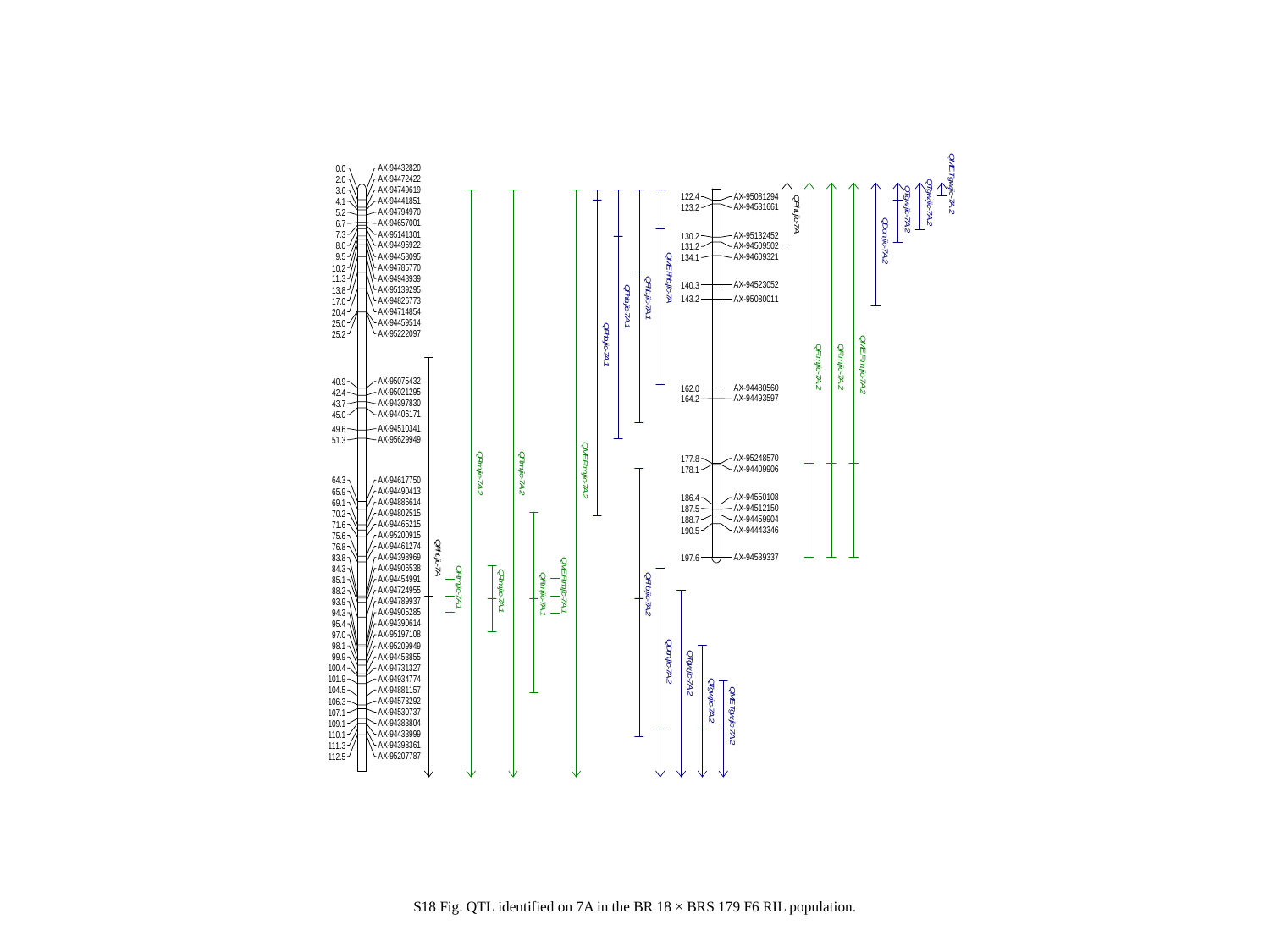

S18 Fig. QTL identified on 7A in the BR 18 × BRS 179 F6 RIL population.

Supplement: S18 Fig — (PPTX) [file pone.0248184.s018.pptx]
